# Mitigation of helium irradiation-induced brain injury by microglia depletion

**DOI:** 10.1186/s12974-020-01790-9

**Published:** 2020-05-19

**Authors:** Barrett D. Allen, Amber R. Syage, Mattia Maroso, Al Anoud D. Baddour, Valerie Luong, Harutyun Minasyan, Erich Giedzinski, Brian L. West, Ivan Soltesz, Charles L. Limoli, Janet E. Baulch, Munjal M. Acharya

**Affiliations:** 1 Department of Radiation Oncology, University of California, Irvine, CA USA; 2 Department of Neurosurgery, Stanford University, Stanford, CA USA; 3 Plexxikon Inc., Berkeley, CA USA

**Keywords:** Space irradiation, Cosmic radiation, PLX5622, Microglia, Cognitive function, Inflammation, Neuron morphology, Spine density, PSD-95, Electrophysiology

## Abstract

**Background:**

Cosmic radiation exposures have been found to elicit cognitive impairments involving a wide-range of underlying neuropathology including elevated oxidative stress, neural stem cell loss, and compromised neuronal architecture. Cognitive impairments have also been associated with sustained microglia activation following low dose exposure to helium ions. Space-relevant charged particles elicit neuroinflammation that persists long-term post-irradiation. Here, we investigated the potential neurocognitive benefits of microglia depletion following low dose whole body exposure to helium ions.

**Methods:**

Adult mice were administered a dietary inhibitor (PLX5622) of colony stimulating factor-1 receptor (CSF1R) to deplete microglia 2 weeks after whole body helium irradiation (^4^He, 30 cGy, 400 MeV/n). Cohorts of mice maintained on a normal and PLX5622 diet were tested for cognitive function using seven independent behavioral tasks, microglial activation, hippocampal neuronal morphology, spine density, and electrophysiology properties 4–6 weeks later.

**Results:**

PLX5622 treatment caused a rapid and near complete elimination of microglia in the brain within 3 days of treatment. Irradiated animals on normal diet exhibited a range of behavioral deficits involving the medial pre-frontal cortex and hippocampus and increased microglial activation. Animals on PLX5622 diet exhibited no radiation-induced cognitive deficits, and expression of resting and activated microglia were almost completely abolished, without any effects on the oligodendrocyte progenitors, throughout the brain. While PLX5622 treatment was found to attenuate radiation-induced increases in post-synaptic density protein 95 (PSD-95) puncta and to preserve mushroom type spine densities, other morphologic features of neurons and electrophysiologic measures of intrinsic excitability were relatively unaffected.

**Conclusions:**

Our data suggest that microglia play a critical role in cosmic radiation-induced cognitive deficits in mice and, that approaches targeting microglial function are poised to provide considerable benefit to the brain exposed to charged particles.

## Introduction

As NASA continues to plan for deep space missions, the potential harmful effects of the space radiation environment on the central nervous system (CNS) functionality have received increased scrutiny. A wealth of data from a number of labs have now documented an impressive array of adverse neurocognitive outcomes following exposure to a variety of radiation types and exposure paradigms [1–9]. Findings from many different rodent models subjected to space relevant, low dose, whole body radiation exposures find long-lasting cognitive impairments [3]. The persistence of these detrimental effects coincides with alterations in biochemical [10–14], molecular [15, 16], cellular [2, 17, 18], structural [1–3, 19], and electrophysiological processes [3, 20–22] that point to the pleiotropic effects of charged particle exposures on the brain. Of particular note, is the persistent inflammatory footprint caused by radiation exposure involving a persistent elevation of “primed” or “activated” microglia.

As the principal immune cells of the CNS microglia represent ~ 12% of all CNS cell types and respond to injury, infection or disease to eliminate accumulated debris thereby serving a neuroprotective role [23, 24]. Recent data have shown microglia to be dependent on colony-stimulating factor 1 receptor (CSF1R) signaling for their survival [25]. CSF1R signaling is critical for early brain development [26], and in healthy adult brains, microglia are the principle cell type expressing CSF1R. Pharmacological CSF1R inhibition elicits a rapid and extensive depletion of microglia in the adult brain [25, 27–29], that can be reverted by removal of CSF1R inhibition. Previous studies also showed that microglia depletion does not induce cognitive impairments [25, 27] and a recent report has demonstrated the potential therapeutic benefits of microglial replacement in the aged brain [30]. Elimination of “old” microglia through CSF1R blockade and subsequent repopulation rejuvenated the microglial phenotype and promoted reversal of age-related changes in cognition, dendritic spine densities, neurogenesis, synaptogenesis, and long-term potentiation [30]. This and other works have clearly pointed to the potential benefits of promoting microglial turnover in the aged or injured brain, and point to the importance of reducing the yield of chronically activated microglia that appear to perpetuate chronic inflammatory signatures in the irradiated brain.

An adaptation of the foregoing approach has demonstrated that transient microglia depletion could forestall cognitive impairments resulting from space-relevant, helium ion (^4^He) exposure [18]. A strength of this approach was the temporary CSF1R blockade using a high-affinity inhibitor, PLX5622, formulated in mouse chow (afforded by an equivalent PLX5622 diet). However, mice were scrutinized using only two behavior tasks, which presents certain limitations. Our past data have shown the lasting effects of charged partial irradiation on cognitive function using six independent behavior tasks [1–3] where improvements on either neurocognitive or neuroinflammation endpoints from 6 weeks to 52 weeks post-exposure were not observed. To more thoroughly evaluate the beneficial neurocognitive and anti-inflammatory effects of PLX5622 against low dose (30 cGy) ^4^He ion irradiation, mice underwent a longer-term PLX5622 administration regimen and were subjected to an extensive battery of seven behavioral tasks 4 to 6 weeks post-irradiation. This testing platform provided a rigorous assessment of cognitive function that was not overly reliant on a single task, cognitive domain, or specific brain region. Our data corroborate much of the past work performed using multiple injury models [27, 31], including cosmic radiation exposure [18], and show marked neurocognitive benefits of CSF1R blockade in the irradiated brain. Although microglial elimination improved cognition when evaluated 4 to 6 weeks following ^4^He exposure, other morphologic, synaptic, and electrophysiological properties of neurons showed relatively subtle or no rescue (i.e., mitigation) effects, suggesting other undefined, possibly more prominent neural targets or effects of CSF1R blockade.

## Materials and methods

### Animals, irradiation and CSF1R inhibitor treatment

**Fig. 1.**
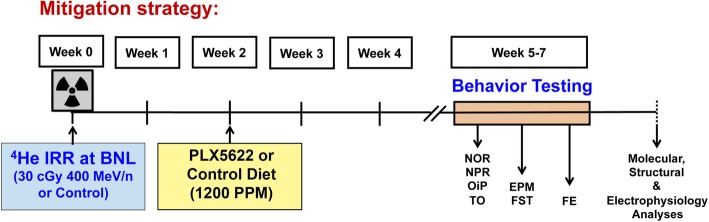
Schematic of the experimental design. 6-months old adult male mice (C57Bl6/J and Thy1-EFGP^+^) received whole body exposure to the ^4^He particles (400 MeV/n, 30 cGy at the Brookhaven National Laboratory). Control (0 Gy) and irradiated (30 cGy) mice received either control chow or PLX5622 chow (1200 PPM) beginning from two-week post-irradiation and continued throughout the study. Mice were administered hippocampal- and frontal cortex-dependent cognitive testing four-week post-irradiation including novel object recognition (NOR), novel place recognition (NPR), object in place (OiP), temporal order (TO), elevated plus maze (EPM), forced swim test (FST) and, in the end, fear extinction memory (FE) tasks. A separate cohort of mice was used for the electrophysiology measurements. After completion of behavior testing, mice were euthanized and tissues were collected for the immunohistochemistry and neuron structure analyses

All animal experimentation procedures described in this study are in accordance with the guidelines provided by NIH and approved by the University of California Irvine, Stanford University and Brookhaven National Laboratory (BNL) Institutional Animal Care and Use Committees. Six-month-old male transgenic mice harboring the Thy1-EGFP transgene were used for the neuron structure analyses in this study (strain Tg(Thy1-EGFP) MJrsJ, stock no. 007788, The Jackson Laboratory, Sacramento, CA). Mice were bred and genotyped to confirm the presence of the Thy1-EGFP transgene. For all other behavioral, molecular, and electrophysiological analyses, 6-month-old wild-type, male mice were used (C57Bl/6J, stock no. 000664, Jackson). Animals were maintained in sterile housing conditions (20 °C ± 1 °C; 70% ± 10% humidity; 12 h/12 h light and dark cycle) and had ad libitum access to standard rodent chow and water. For all studies, the mice were divided into 4 experimental groups (*N* = 8–10 mice per group): unirradiated and irradiated receiving control chow (0 Gy + Con chow and 30 cGy + Con chow), and unirradiated and irradiated receiving CSF1R inhibitor chow (0 Gy + PLX5622 and 30 cGy + PLX5622; Fig. 1). During irradiation, mice were loosely restrained in Lucite containers with breathing holes (3 × 1.5 × 1.5 in.) for exposure to 30 cGy of 400 MeV/n ^4^He particles (dose rate = 15–25 cGy/min) at the NASA Space Radiation Laboratory (NSRL) at Brookhaven National Laboratory (Long Island, NY). The Physics Dosimetry Group of the NSRL provided beam characterization and dosimetry. Concurrent control mice were placed in Lucite boxes at the NSRL for the same length of restraint time as required for the irradiation. An average duration of ^4^He particle exposure was 102 ± 1.63 s with the dose rate of 17.78 ± 0.13 cGy per min (Mean ± SEM, *N* = 6 runs). Two weeks after irradiation mice were provided control or PLX5622 chow. CSF1R inhibitor, PLX5622, was provided by Plexxikon (Berkeley, CA) and formulated in AIN-76A standard chow by Research Diets (New Brunswick, NJ) at a dose of 1200 PPM. Control mice received AIN-76A chow without PLX5622. All mice were maintained on their respective PLX5622 or control diet from 2 weeks post-irradiation through the duration of all studies.

### Behavioral testing

To determine the effects of microglia elimination on cognitive function after irradiation, mice were subjected to behavioral testing 1 month later. Testing was conducted over 3 to 4 weeks and included four open field, spontaneous exploration tasks: novel object recognition (NOR), object in place (OiP), temporal order (TO) and novel place recognition (NPR). Measures of anxiety- and depression-like behaviors were assessed by elevated plus maze (EPM) and forced swim test (FST) respectively. To minimize repeat testing-related fatigue, NPR, EPM, and FST were conducted on separate cohorts of mice. Time spent exploring both familiar and novel place or object was counted when the nose of the mouse was within 1 cm and pointed in the direction of the object. Mice did not show object climbing or neophobic behavior. NOR, OiP, TO, and NPR data are presented as a discrimination index (DI) and calculated as ([Novel location exploration time/Total exploration time] − [Familiar location exploration time/Total exploration time]) × 100 where a positive index indicates that a mouse spent more time exploring novelty (i.e., switched objects or locations), while a negative score indicates little or no preference for exploring novelty. EPM data are presented as the percent of total time spent in the open arms of the EPM. FST data are presented as the percent of total time spent immobile/floating over the trial duration for each mouse. All of these behavioral testing paradigms followed our previously described protocols [1, 2, 15, 29]. Behavior data analysis was conducted independently and blind and presented as the average of all trials scored for each task.

Last, a separate cohort of mice (*N* = 8 per group) underwent hippocampus-dependent fear extinction (FE) testing to determine whether mice could learn and later extinguish conditioned fear responses, using a series of established fear extinction assays [32]. Testing occurred in a behavioral conditioning chamber (17.5 × 17.5 × 18 cm, Coulbourn Instruments) with a steel slat floors (3.2 mm diameter slats, 8 mm spacing). Throughout the conditioning, extinction training and testing phases, the bottom acrylic collection plate was scented with a spray of 10% acetic acid in water. For the initial fear conditioning phase (day 1), mice were allowed to habituate to the chamber for 2-min. Three pairings of an auditory conditioned stimulus (16 kHz tone, 80 dB, lasting 120 s; CS) co-terminating with a foot shock unconditioned stimulus (0.6 mA, 1 s; US) were presented at 2-min intervals. On the following 3 days of extinction training, mice were initially habituated to the same context for 2-min before being presented with 20 non-US reinforced CS tones (16 kHz, 80 dB, lasting 120 s, at 5 s intervals). On a final day of fear testing, mice were presented with only three non-US reinforced CS tones (16 kHz, 80 dB, lasting 120 s) at a 2-min intervals in the same context. Freezing behavior was recorded with a camera mounted above the chamber and scored by an automated measurement program (FreezeFrame, Coulbourn Instruments).

### Immunohistochemistry

Following behavioral testing (7–8 weeks post-irradiation), animals were deeply anesthetized with isoflurane and euthanized with saline with heparin (10 U/ml, Sigma-Aldrich) followed by 4% paraformaldehyde (intracardiac perfusion). Brains were cryoprotected (30% sucrose) and sectioned coronally (30 μm thick) using a cryostat (Leica Microsystems, Germany). For each endpoint 3–4 representative coronal brain sections from each of 4–6 animals per experimental group were selected through the middle of hippocampus (2.10 to 2.95 mm from bregma) at approximately 15 section intervals and stored in tris-buffered saline (TBS, 100 mM, pH 7.4, Sigma-Aldrich, St. Louis, MO). For the immunofluorescence analyses, the following primary and secondary antibodies were used: rabbit anti-IBA-1 (1:500, Wako), rat anti-mouse CD68 (1:200, AbD Serotec), mouse anti-NG2 (1:400, Millipore), mouse anti-GFAP (1:500, Millipore), donkey anti-rabbit or anti-mouse conjugated with Alexa Fluor 488, 568 or 647 (1:400, Life Technologies/Invitrogen). For the immunofluorescence labeling of PSD-95 mouse anti-PSD-95 (Thermo Scientific; 1:1000) primary antibody was used with Alexa Fluor 594 secondary antibody (1:1000). Tissues were DAPI nuclear counterstained (15 μM final concentration in TBS) and sealed in gold slow fade/antifade mounting medium (Life Technologies). IBA-1^+^ and GFAP^+^ cells were visualized under fluorescence as green; CD68^+^ and NG2^+^ cells, and PSD-95 immunoreactivity as red; nucleus as blue.

Immunofluorescent sections were imaged using Nikon Eclipse Ti C2 microscope to obtain 20 to 30 z stacks (1024 × 1024 pixels, 0.5 to 1 μm each) using 10 and 60× PlanApo oil-immersion lens (Nikon). For quantification of glial cells, 3D deconvolution and reconstruction was carried out using the AutoQuantX3 algorithm (MediaCybernetics). Deconvolution combined with 3D reconstruction yields higher spatial resolution images for the immunofluorescent cell bodies and stellae ([29, 33]). Quantification was facilitated using Imaris filament and spot tool (v8.0, Bit Plane Inc., Switzerland) that detect immunostained puncta within 3D deconvoluted image stacks based on a predefined diameter and red/green channel intensity threshold. Glial cells data are expressed as mean immunoreactivity (percentage) relative to unirradiated controls (0 Gy + Con chow).

### Confocal imaging, neuronal morphometry and spine parameters, electrophysiology

The expression of EGFP in specific subsets of neurons provides for the high-resolution imaging and quantification of neuronal structure. In previous studies, we demonstrated that cosmic radiation exposures reduced dendritic complexity of the mature neurons. Here, we have conducted morphometric analyses of neurons in the hippocampal granule cell and CA1 pyramidal cell layers at 4 weeks post-irradiation, using the same rigorously defined morphometric and experimental criteria ([1, 34]. Briefly, all morphometric parameters and spine density were quantified from reconstructed neurons in a region of interest (1.2 × 1.2 mm^2^) of the hippocampal DG and CA1 subregions (2.10 to 2.95 mm from bregma) cut at a thickness of 100 μm for confocal imaging. Three sections per animal were used to generate representative z stacks from 3–4 animals using a Nikon Eclipse TE 2000-U microscope (Nikon, Japan). Quantification included the dendritic structure of both apical and basal dendrites of GCL and CA1 pyramidal neurons. An algorithm for tracing dendritic filaments was used to reconstruct the entire dendritic tree, where tracing originates from the soma and terminates near terminal dendritic diameter thresholds. Reconstructed dendritic segments can be analyzed under higher magnification for dendritic spines that can be labeled, manually verified, morphologically categorized, and quantified. All morphometric parameters were validated from an independent series of pilot reconstructions in both manual and semiautomatic modes. Images were then compared for accuracy and consistency to ensure that selected parameters represented actual variations in dendritic structure. Parameters of neuronal structure that were identified and quantified through image reconstruction and deconvolution using the Imaris software suite (Bitplane Inc., Switzerland) included the cell body, dendritic and axonal length, branching and branch points, dendritic complexity, spines, and boutons. The number of dendritic spines was determined by summing total number of spines in the same region of interest, where spine density was calculated by dividing total dendritic length by the total number of spines. Spines were classified based on the following morphological parameters [35–37]: (i) stubby spine: stubby spines are devoid of a neck, diameter of the head is almost equal to the total length of the spine. (ii) Long-thin spine: length of neck is greater than its diameter and the head is clearly distinguishable but has a diameter less than the length of the neck. (iii) Mushroom spine: mushroom spines have a large head and a narrow neck, diameter of the head is greater than the width of the neck. (iv) Filopodia spine: total spine length is greater than 1 μm with the complete absence of a head. Electrophysiological measurements followed methods as described previously [3]

### Statistical analyses

The level of significance for behavioral testing was assessed by two-way or repeated-measures two-way ANOVA as applicable, along with Bonferroni’s multiple comparison using Prism data analysis software (v6.0). Data are expressed as mean ± standard error of the mean. Statistical significance was assigned at P<0.05.

## Results

### PLX5622 treatment mitigates ^4^He irradiation-induced behavioral impairments

**Fig. 2:**
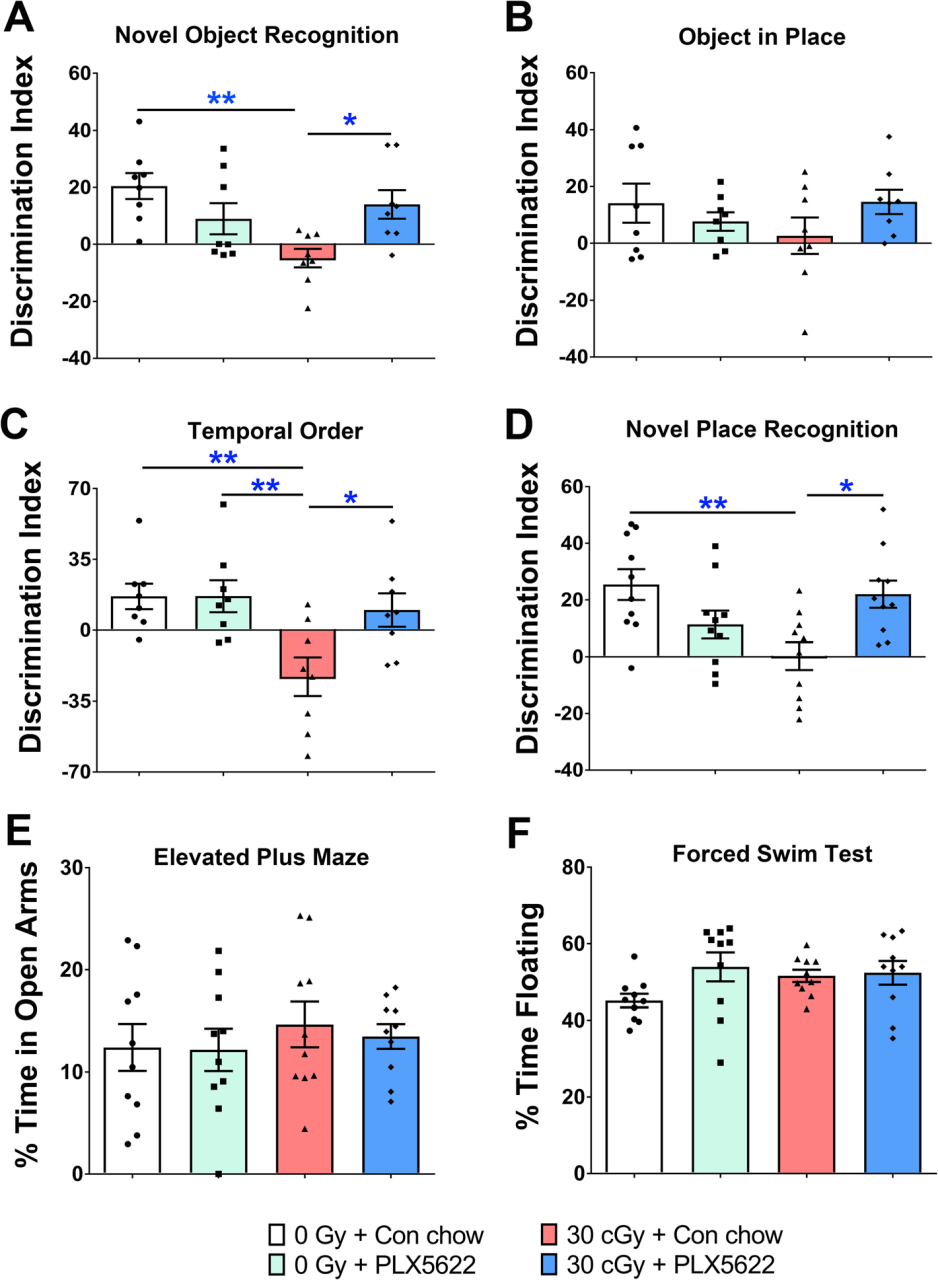
Treatment with PLX5622 mitigates ^4^He irradiation-induced cognitive dysfunction. One-month post-irradiation and PLX5622 treatment, mice were tested on the hippocampus- and frontal cortex-dependent cognitive function tasks. The tendency to explore novel location(s) or object was determined by the Discrimination index, calculated as ([Novel location exploration time/Total exploration time] − [Familiar location exploration time/Total exploration time]) × 100. **a–f** Behavior of control mice receiving PLX5622 treatment (0 Gy + PLX5622) was indistinguishable from the unirradiated control mice (0 Gy + Con chow) as indicated by no significant difference between discrimination indices (**a–d**), time spent in open arms (**e**), or percent time floating (**f**). **a**, **c**, **d** Exposure to ^4^He particles (30 cGy + Con chow) show significant behavior deficits on the novel object recognition (NOR, **p < 0.01), temporal order (TO, **p < 0.01) and novel place recognition (NPR, **p < 0.01) tasks. **b** The effect of either ^4^He particles or PLX5622 was not significant for the object in place task (OiP). **a**, **c**, **d**^4^He-irradiated mice receiving PLX5622 treatment showed significant improvements on the performance on NOR (*p < 0.05), NPR (*p < 0.05), and TO (*p < 0.05) tasks. **e**, **f** There was no significant differences between groups for the anxiety (elevated plus maze, EPM) and depression-like behavior (forced swim test, FST). Data are presented as mean ± SEM (**a–c***n* = 8/group; **d–f***n* = 10/group, **a**). *p* values are derived from two-way ANOVA and Bonferroni’s multiple comparisons test. *p < 0.05 and **p < 0.01 compared with the 30 cGy + Con chow group

One-month post-irradiation and PLX5622 treatment animals were habituated and tested on the NOR, OiP, TO, and NPR open arena tasks. For each of these tasks, the total exploration of objects during the familiarization phase did not differ among the 4 experimental groups (Supplementary Tables S1, S2, S3, S4). After the 5-min retention interval, following familiarization, the test phase of the NOR showed a significant overall group difference between the cohorts for the discrimination index (*F*_(3,28)_ = 5.35, *P* = 0.005). Irradiated mice on control chow (30 cGy + Con chow) showed a significantly reduced preference for the novel object compared to control mice on control chow and to irradiated mice administered the PLX5622 diet (Fig. 2a; 0 Gy + Con chow and 30 cGy + PLX5622; *P* = 0.004 and 0.046, respectively). Additionally, we found a significant interaction for the radiation effect (*F*_(1, 28)_ = 4.76, *P* = 0.004). No significant overall group differences were detected between the cohorts in the OiP testing (Fig. 2b; *F*_(3,28)_ = 1.11, *P* = 0.36).

Significant overall group differences were found between the cohorts for the discrimination index on the TO task (Fig. 2c; *F*_(3,28)_ = 5.51, *P* = 0.004) and for NPR (Fig. 2d; *F*_(3,36)_ = 5.17, *P* = 0.005). Irradiated mice on control chow (30 cGy + Con chow) showed a significantly reduced preference for novel location (NPR) compared to control mice on control chow and to irradiated mice administered the PLX5622 diet (Fig. 2d; 0 Gy + Con chow and 30 cGy + PLX5622; *P* = 0.007 and 0.024, respectively). While an interaction effect was found between irradiation and PLX5622 treatment (*F*_(1, 36)_ = 7.71, *P* = 0.008 and, *F*_(1, 36)_ = 4.92, *P* = 0.04, respectively), the drug treatment was able to mitigate the adverse effects of irradiation. For the TO task, the mice exposed to 30 cGy of ^4^He particles also exhibited significant reductions in the preference to explore novelty as compared to all other cohorts (Fig. 2c; 0 Gy + Con chow, 0 Gy + PLX5622 and 30 cGy + PLX5622; *P* = 0.010, *P* = 0.010, and *P* = 0.046, respectively). In addition, a significant interaction for the radiation effect was found (*F*_(1, 28)_ = 8.23, *P* = 0.008). Together, these data demonstrate that microglia depletion following a space-relevant radiation exposure prevents impairments in perirhinal cortex and hippocampal-dependent behavior as reflected in reductions in appreciation for novelty during spatial exploration and recency memory behavior testing. Moreover, the behavior of control mice receiving PLX5622 did not differ significantly from the control mice receiving vehicle and, therefore, reiterated our previous findings [29] that microglia depletion in control mice has no adverse impact on cognitive function.

Radiation exposure has also been shown to adversely impact mood including anxiety- and depression-like behaviors in mice [2, 3, 38]. To assess anxiety-like behavior mice were evaluated on the EPM, where animals exhibiting increased anxiety spend more time in the close arms and less time in the open arms of the maze [39]. In this testing paradigm, at 6 weeks post-irradiation, ^4^He particles and PLX5622 diet elicited no differences between the groups in time spent in the open arms of the EPM (Fig. 2e; *F*_(3,36)_ = 0.32, *P* = 0.81). Similarly, analysis of depression-like behavior on the FST, where depression is measured by the time spent immobile and floating as compared to swimming, demonstrated no overall group effect on time spent floating (Fig. 2F; *F*_(3,36)_ = 2.03, *P* = 0.13). These findings suggest that anxiety- and depression-like behaviors do not manifest at early (6–8 weeks) post-exposure times using 30 cGy of ^4^He particles and are not affected by microglia depletion.

**Fig. 3:**
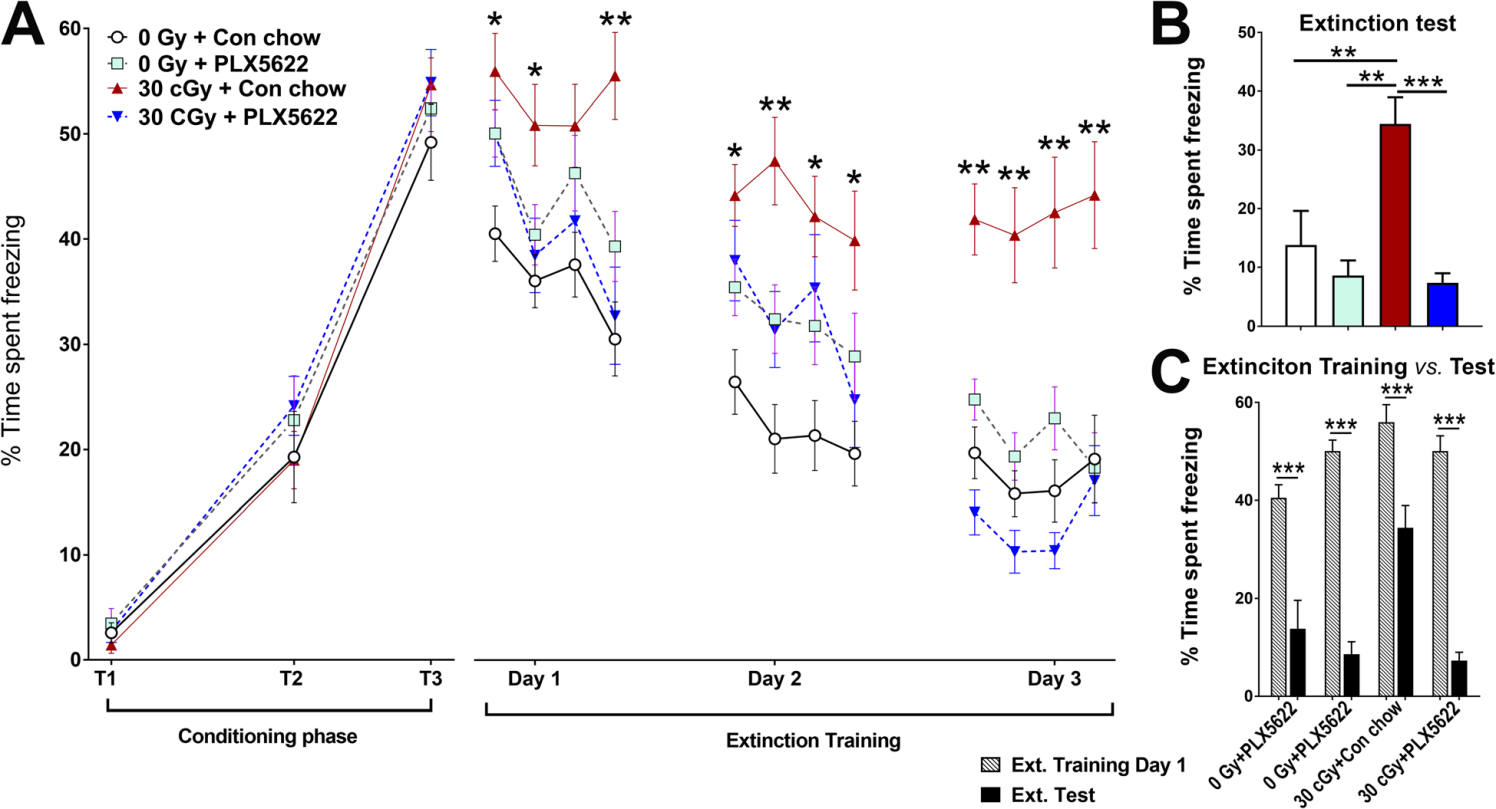
PLX5622 treatment ameliorates ^4^He irradiation-induced, hippocampal-dependent, fear extinction memory impairments. **a** During the conditioning phase, all mice showed elevated freezing following a series of three tone-shock parings (80 dB, 0.6 mA, T_1_–T_3_). 24 h after the conditioning phase, fear extinction training was administered every 24 h (20 tones) for 3 days in the same environment (spatial and odor context). All mice showed a gradual decrease in the freezing behavior (day 1–3) except ^4^He-irradiated mice (30 cGy + Con chow) which showed a significantly higher time in freezing compared to other groups (0 Gy + Con chow, 0 Gy + PLX5622 and 30 cGy + PLX5622; *p < 0.01; **p < 0.001). **b** During the extinction test (24 h after extinction training), control mice receiving vehicle or drug treatment (0 Gy + Con chow and 0 Gy + PLX5622) showed abolished fear memory compared to ^4^He exposed mice given vehicle (30 cGy + Con chow, *p < 0.01). In contrast, the freezing levels for the ^4^He-irradiated mice receiving PLX5622 were comparable to the control mice (0 Gy + Con chow). **c** Comparison of percentage time freezing during the first day of extinction training phase versus extinction test day show significant differences for each experimental cohort. Data are presented as mean ± SEM (*n* = 8 per group, **a**). *p* values are derived from repeated-measures two-way ANOVA and Bonferroni’s multiple comparisons test. *p < 0.05 and **p < 0.01 compared with the 30 cGy + Con chow group (**a**, **b**); ***p < 0.0001 compared with percentage freezing (first five tones) during the extinction training day 1 (**c**)

Exposure to space-relevant radiation has also been demonstrated to impair dissociative learning [2]. As such, fear extinction testing was used to determine whether mice exposed to ^4^He particles could learn and subsequently extinguish conditioned fear responses [32]. During the conditioning phase of FE testing, all groups of mice exhibited comparable associative learning as demonstrated by similar times spent freezing during the tone-shock conditioning phase (Fig. 3; T_1_–T_3_; 49 to 55%, T_3_). A significant overall group × extinction training phase interaction effect as determined by repeated measures two-way ANOVA for percentage of time spent freezing during each training day (*F*_(42, 1956)_ = 1.49, *P* = 0.02). During the subsequent extinction training days, where mice were presented with 20 tones per day (5 s intervals) in the same context as the conditioning phase with no foot shock, the irradiated mice on control chow (30 cGy + Con chow) continued to show increased freezing as compared to the irradiated mice administered PLX6522 chow (30 cGy + PLX5622) and as compared to both control cohorts (0 Gy + Con chow and 0 Gy + PLX5622, *P* = 0.01 and *P* = 0.001 respectively). These data indicate that PLX5622 treatment to the irradiated animals prevented impairments in the ability to dissociate the learned response (freezing) to a prior aversive event (tone-shock pairing). Twenty-four hours after completion of extinction training, the mice underwent extinction testing where they were presented with just 3 tones (120 s intervals) in the same testing environment as used for extinction training. While an interaction effect was found between irradiation and PLX5622 treatment (*F*_(1, 28)_ = 5.92, *P* = 0.02 and *F*_(1, 28)_ = 16.51, *P* = 0.0004, respectively), the drug treatment was again able to mitigate the adverse effects of irradiation (Fig. 3b). Irradiated mice receiving the control chow (30 cGy + Con chow) demonstrated an inability to abolish fear memories during this retrieval testing and again exhibited increased freezing and the impairment in fear extinction was prevented by microglia depletion with PLX5622 (Fig. 3b; 0 Gy + Con chow, 0 Gy + PLX5622 and 30 cGy + PLX5622; *P* = 0.003, *P* = 0.0003, and *P* = 0.0001, respectively). In addition, two-way ANOVA for the percentage time spent freezing during the first day of extinction training and extinction test phases revealed significant interactions for experimental treatments (Fig. 3c; *F*_(3, 156)_ = 11.64, *P* < 0.0001). Moreover, significant differences between extinction training and testing phases were found for each experimental groups (*F*_(1, 156)_ = 179, *P* < 0.0001). This hippocampus-dependent FE testing paradigm provides a relative invasive measure of elevated anxiety and impairments in associative/dissociative learning, and demonstrate that exposure to 30 cGy of ^4^He particles induces impairments similar to a post-traumatic stress disorder that can be abolished by PLX5622-mediated depletion of microglia.

### PLX5622 reduced microglia in the control and ^4^He particle exposed brains

**Fig. 4:**
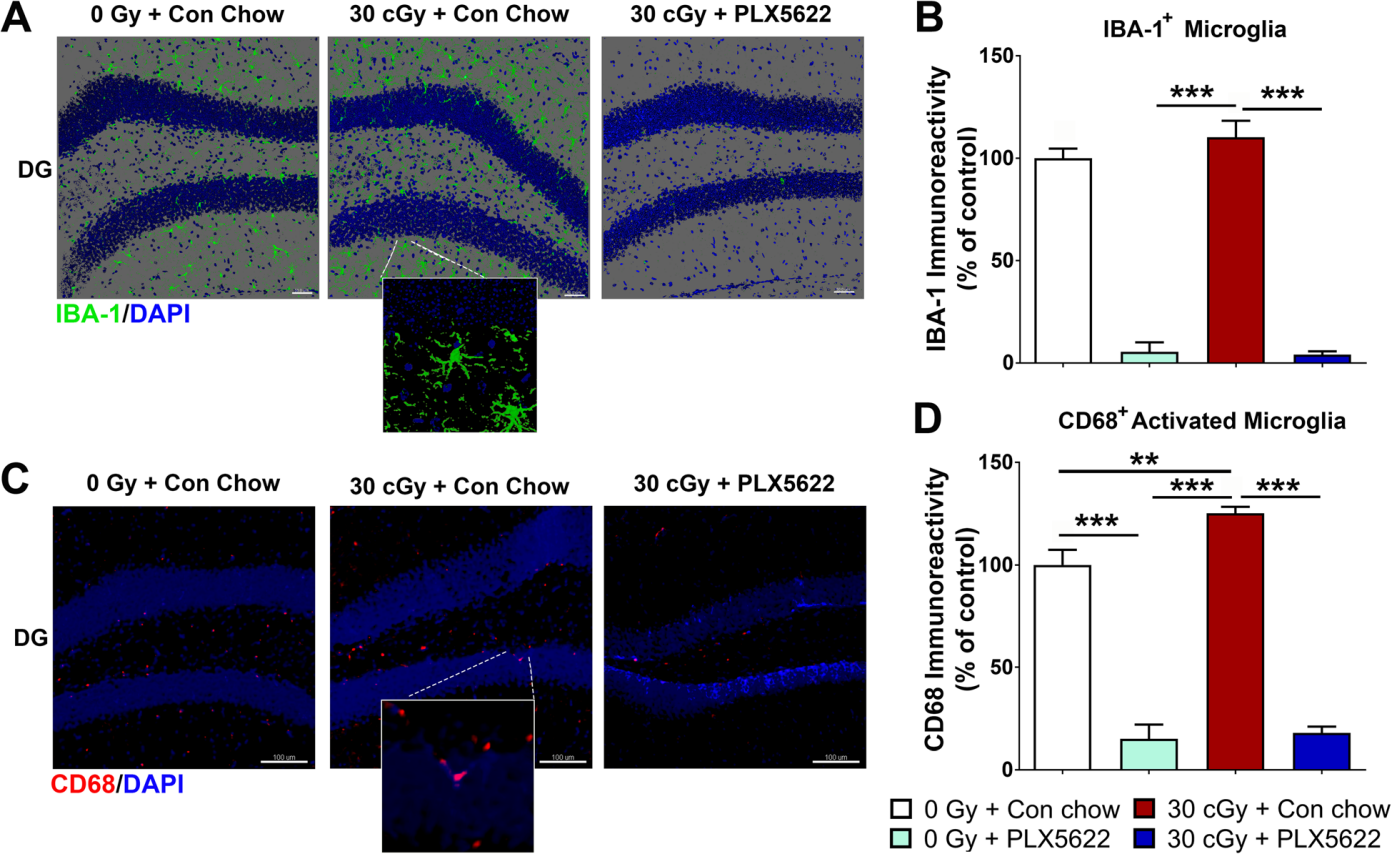
Treatment with PLX5622 depletes microglia from the control and ^4^He-irradiated brains. **a**, **b** Immunofluorescence staining, confocal microscopy and 3D algorithm-based volumetric quantification of pan microglial marker IBA-1 (green; nuclear counter stain, DAPI, blue) show > 90% depletion of microglia in the control (0 Gy + PLX5622) and ^4^He-irradiated (30 cGy + PLX5622) hippocampus at 8-week post-PLX5622 treatments (***p < 0.001 compared to 0 Gy + Con chow and 30 cGy + Con chow). ^4^He irradiation did not alter the IBA-1 immunoreactivity (0 Gy + Con chow vs. 30 cGy + PLX5622). **c**, **d** Exposure to ^4^He particles elevated microglial activation maker, CD68, immunoreactivity (***p < 0.001 compared to 0 Gy + Con chow). Treatment with PLX5622 for 8 weeks eliminates 80–90% of CD68^+^ activated microglia (red; nuclear counter stain, DAPI, blue) in the control (0 Gy + PLX5622) and ^4^He-irradiated (30 cGy + PLX5622) hippocampus (**p < 0.001 compared to 0 Gy + Con chow and 30 cGy + Con chow). Data are presented as mean ± SEM (*n* = 4–6/group). *p* values are derived from two-way ANOVA and Bonferroni’s multiple comparisons test. Scale bar: 50 μm, **a** 100 μm, **b** 5 μm, inserts, **a**, **b**

Our past reports have clearly demonstrated that space radiation-induced microglial activation contributes to cognitive impairments [2, 3]. To determine the effectiveness of CSF1R inhibition via PLX5622 in the current study, the number of IBA-1^+^ and CD68^+^ activated microglia was quantified (Fig. 4). Both control and irradiated mice administered the PLX5622 diet showed a significant depletion in the number of IBA-1^+^ microglial cells after ~ 8 weeks of treatment (Fig. 4a, b). Two-way ANOVA revealed a significant PLX5622 treatment effect for the IBA-1 immunoreactivity (*F*_(1, 20)_ = 429.2, *P* < 0.0001). 3D algorithm-based volumetric quantification (AutoQuant and Imaris) of confocal z stacks showed that CSF1R blockade lead to > 90% reduction in the IBA-1 immunoreactivity in control and ^4^He-irradiated brains (Fig. 4b; *P* < 0.0001 in each case). ^4^He irradiation (30 cGy + Con chow) did not change the IBA-1 immunoreactivity compared to the control brains (0 Gy + Con chow). In contrast, exposure to 30 cGy of ^4^He particles significantly increased the immunoreactivity of an activated microglial marker CD68 (*P* < 0.01, 0 Gy + Con chow versus 30 cGy + Con chow). PLX5622 treatment significantly ablated CD68 immunoreactivity in the control (0 Gy + PLX5622) or irradiated (30 cGy + PLX5622) brains (Fig. 4c, d, *P* < 0.0001; and *P* < 0.001 vs. 0 Gy + Con chow). Two-way ANOVA revealed significant interaction for the radiation and the PLX5622 treatment on CD68 immunoreactivity (*F*_(1, 13)_ = 9.66, *P* < 0.008 and *F*_(1, 13)_ = 454.1, *P* < 0.0001, respectively). These data indicate that ^4^He irradiation-induced microglial activation is, in part, associated with cognitive impairments.

### Effects of ^4^He irradiation, PLX5622 treatment on oligodendrocyte progenitor cells, and astrocytes

**Fig. 5:**
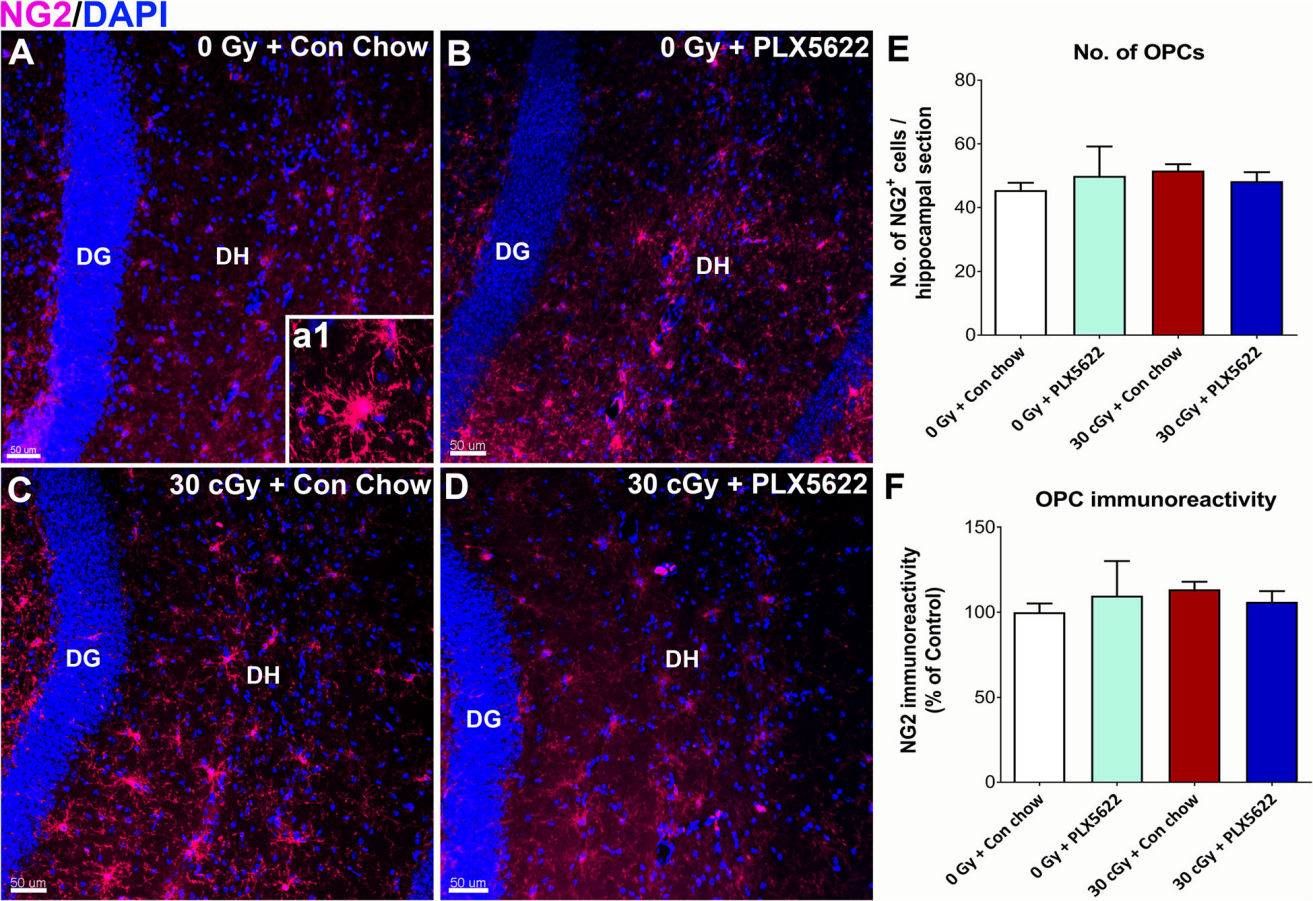
CSF1R inhibitor treatment did not alter oligodendrocyte progenitor cells (OPCs) in the control or ^4^He-irradiated brains. **a–d** and **a1** Representative confocal z stacks for the OPC marker NG2 (magenta; nuclear counter stain, DAPI, blue) is shown for the 0 Gy and 30 cGy irradiated groups receiving either control chow or PLX5622 chow. **e–f** Neither ^4^He irradiation nor PLX5622 treatment alter the number of NG2^+^ cells or the gross immunoreactivity. Data are presented as mean ± SEM (*n* = 4/group). Scale bars: 50 μm, **a–d**; 5 μm **a1**

**Fig. 6:**
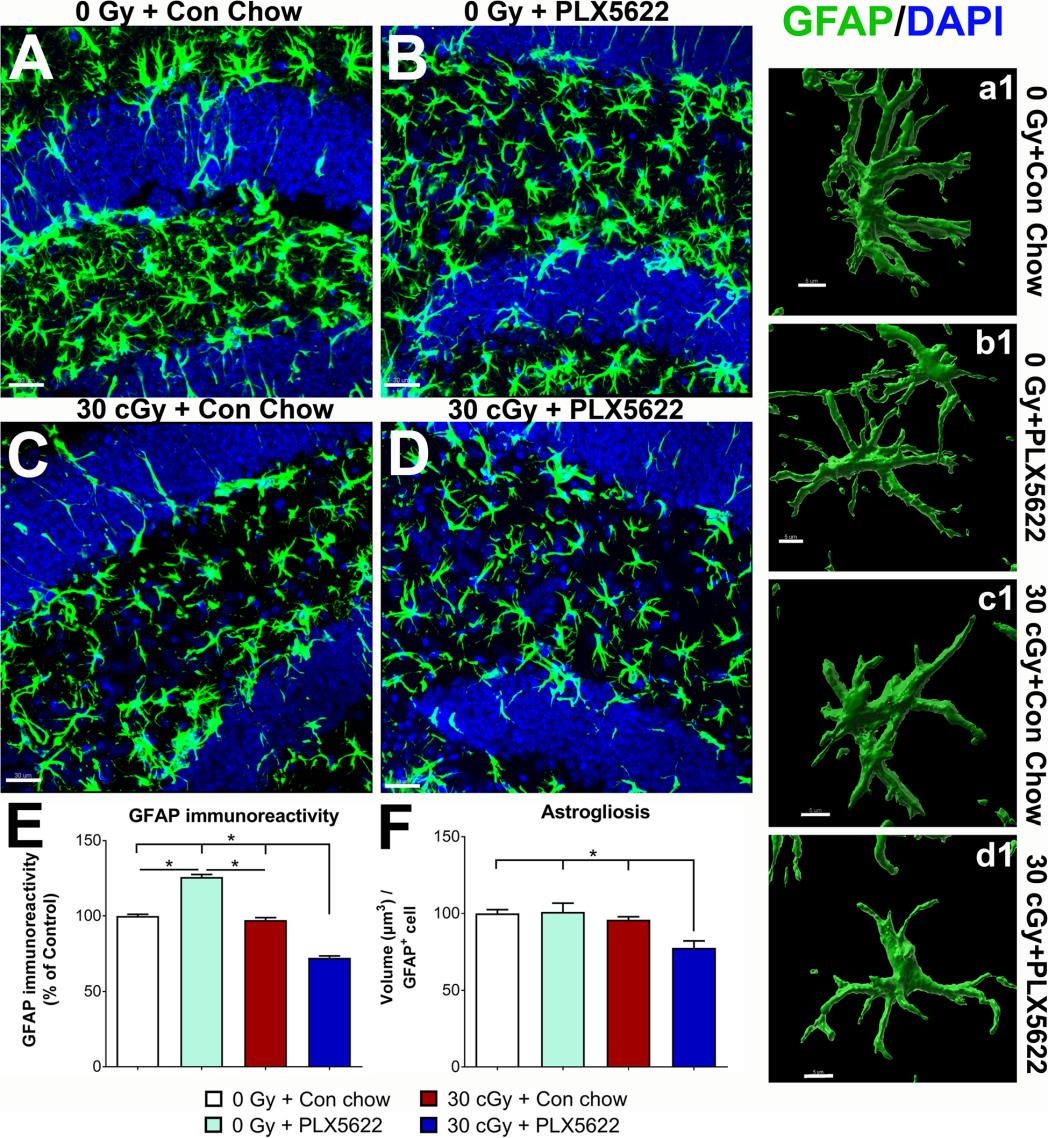
Effect of ^4^He irradiation or PLX5622 treatment on astrogliosis. **a–f** Immunofluorescence staining, confocal microscopy, and 3D algorithm-based volumetric quantification of astrocytic marker GFAP (green; nuclear counter stain, DAPI, blue) show increased gross GFAP immunoreactivity in the control mice receiving PLX5622 (0 Gy + PLX5622) at 8 weeks post-PLX5622 treatments (*p < 0.001 compared to 0 Gy + Con chow and 30 cGy + Con chow). ^4^He-irradiated mice receiving PLX5622 showed decreased GFAP immunoreactivity (*p < 0.001, 30 cGy + PLX5622 compared to all other groups). **a1–d1** Exposure to ^4^He particles and PLX5622 treatment reduced astrogliosis (GFAP volume per cell; *p < 0.001 compared to all other groups). Data are presented as mean ± SEM (*n* = 4/group, **e**; and *n* = 21–24 GFAP^+^ cells/group, **f**). *p* values are derived from two-way ANOVA and Bonferroni’s multiple comparisons test. Scale bars: 30 μm, **a–d**; 5 μm, **a1–d1**

To confirm non-specific effects of PLX5622 treatment on other glial cells, we performed volumetric quantification of oligodendrocyte progenitor cells (NG2^+^) and astrocytes (GFAP^+^) in the control and ^4^He-irradiated brains (Figs. 5 and 6). Neither PLX5622 treatment nor ^4^He irradiation altered NG2 cell number or immunoreactivity (Fig. 5e, f). On the other hand, PLX5622 treatment elevated GFAP gross immunoreactivity in the control brain (0 Gy + PLX5622) compared to 0 Gy and 30 cGy irradiated mice receiving vehicle (*P < 0.001, Fig. 6a–e) Further, 3D algorithm-based volumetric assessment of individual GFAP^+^ astrocyte morphology showed reduced cell volume in the 30 cGy + PLX5622 treatment (Fig. 6F, a1–d1).

**Fig. 7:**
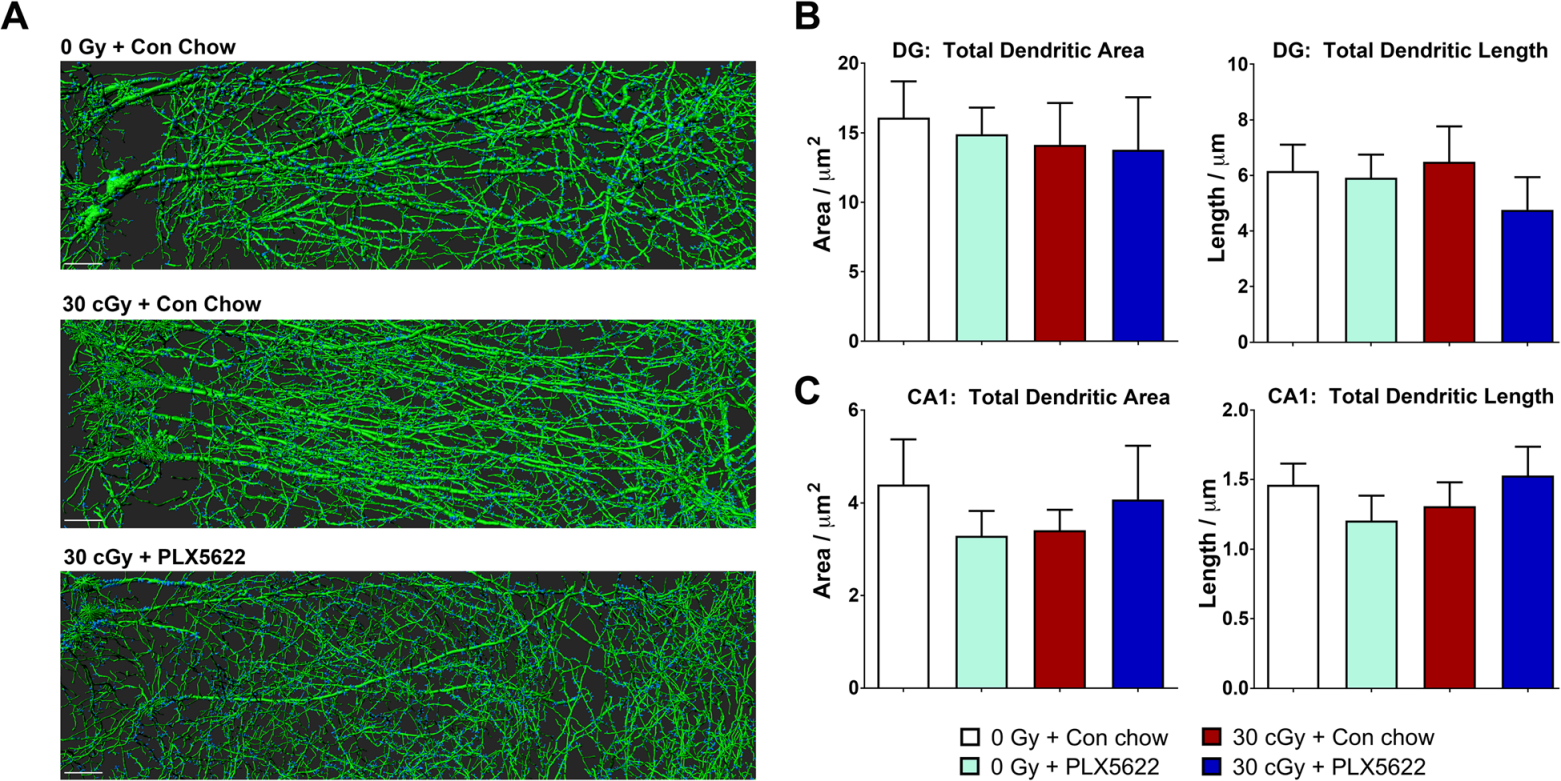
^4^He irradiation or PLX5622 treatment did not alter neuron dendritic parameters. **a** Representative deconvoluted reconstruction of eGFP^+^ CA1 pyramidal neurons showing the basal and apical dendrites (green). **b**, **c** Quantification of total dendritic area and length from the dentate gyrus (DG) granule cell layer (GCL) neuron and CA1 pyramidal neuron did not show a significant difference after 4-weeks between the control (0 Gy) and ^4^He-irradiated (30 cGy) mice treated with PLX5622. Data are presented as mean ± SEM (*n* = 4/group). Scale bar: 5 μm, **a**

### Effects of ^4^He irradiation, PLX5622 treatment on neuron morphology, and synaptic parameters

Our past studies have established the drastic impact of cosmic radiation exposure on mature neuronal morphology and spine density [1, 2]. To determine the effects of exposure to ^4^He particles or PLX5622 on mature neuron structure, hippocampal DG and CA1 sub-regions were analyzed for dendritic tree and spine morphology parameters 4 weeks post-irradiation. We used a transgenic mouse model expressing enhanced green fluorescent protein (eGFP) under the control of a modified Thy1 promoter that restricts expression to certain subsets of neurons. Intense eGFP fluorescence has been reported in retinal ganglion cells, mossy fibers, many cortical neuron layers and hippocampus [40]. eGFP expression in homozygous or hemizygous Thy1-EGFP mice provides a brightly fluorescent signal that expedites the micromorphometric analyses and reveals salient features of a subset of mature neurons. The strong signal-to-noise ratio of eGFP^+^ neurons provides for an accurate, precise, and rigorous analysis and quantification of the dendritic tree, dendritic complexity and fine spine morphology, and that alleviates issues associated with traditional Golgi staining procedures. We did not find a significant effect of either charged particle irradiation or PLX5622 treatment on the DG granule cell or CA1 pyramidal neuron dendritic structure at 4 weeks post-exposure (Fig. 7). These parameters included total dendritic area and length (Fig. 7a–c), and number of dendritic branches and branch points (Supplementary Figure 1). Analysis of spine morphology parameters including the number of spines and spine density (Supplementary Figures 2 and 3) also showed no statistically significant changes induced by radiation or PLX5622.

### Effects of ^4^He irradiation, PLX5622 treatment on dendritic spine volume, and spine morphology

While our past data have shown that cosmic radiation exposure reduced dendritic spine density in the brain [2, 3], this effect was less apparent under the current irradiation conditions. Detailed analysis of dendritic spine volumes did not show significant alterations (Supplementary Figure 3). While the reasons for this remain uncertain, differences in spine turnover rates between different regions of the brain [41] and their susceptibility to cosmic radiation-induced deterioration may provide a partial explanation.

**Fig. 8:**
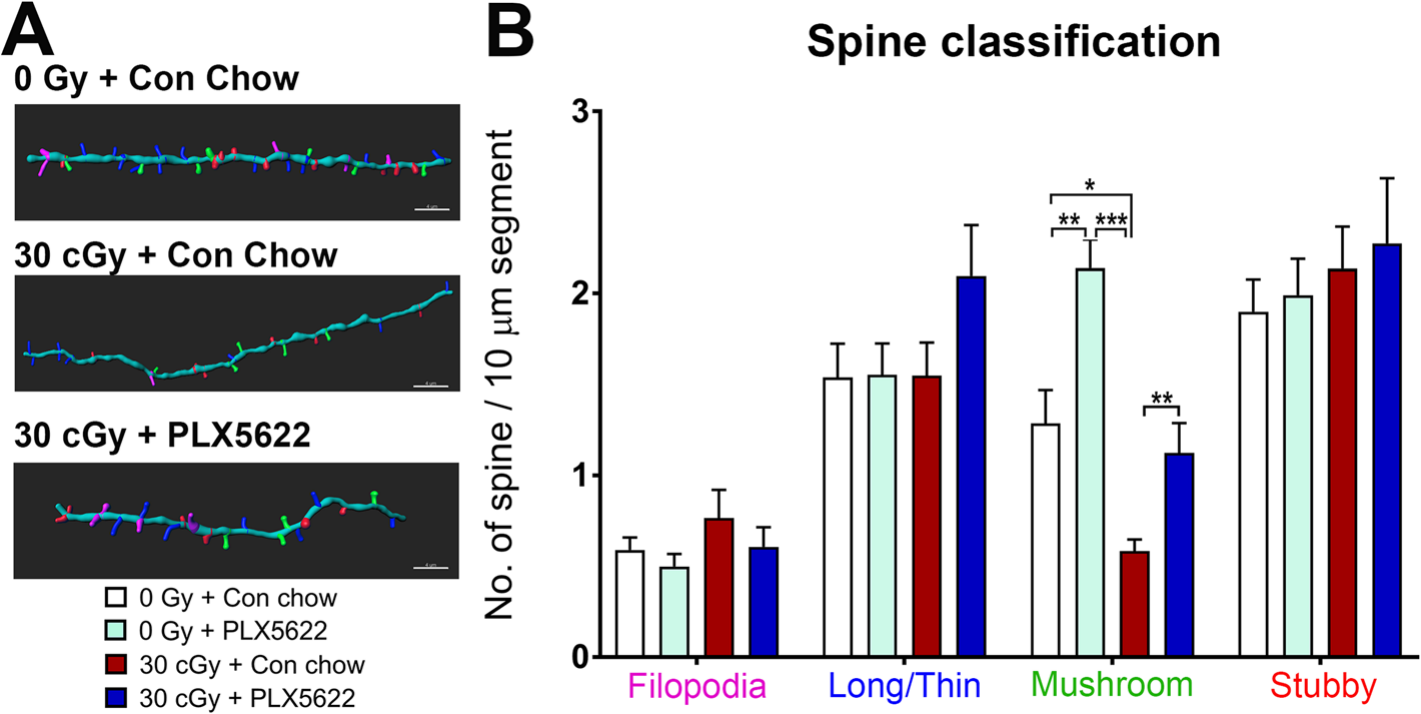
Effect of ^4^He particle exposure or PLX5622 treatment on the number of morphologically distinct spines. **a**, **b** Quantification of reconstructed apical dendritic segments from the CA1 pyramidal neurons show that either exposure ^4^He particles or PLX5622 treatment did not have a significant impact on fliopodia, long-thin, or stubby spine types. Radiation exposure leads to a significant decline in the mushroom spines (*p = 0.025, 0 Gy + Con chow vs. 30 cGy + Con chow). Treatment with PLX5622 significantly improved the number of mushroom spines in the control (**p < 0.01 0 Gy + PLX5622 *vs*. 0 Gy + Con chow) or ^4^He-irradiated dendrites (**p < 0.01 vs. 30 cGy + PLX5622; ***p < 0.002 vs. 0 Gy + PLX5622). Data are presented as Mean ± SEM (*n* = 3–4/group). *p* values are derived from two-way ANOVA and Bonferroni’s multiple comparisons test. *p < 0.025, **p < 0.01, and ***p < 0.002. Dendrite (sky blue), filopodia (magenta), long-thin (blue), mushroom (green), and stubby spines (red). Scale bar, 4 μm, **a**

To further evaluate potential effects of ^4^He irradiation or PLX5622 treatment on the susceptibility of morphologically distinct spines, sub-classes of spines were categorized and quantified using a 3D algorithm-based volumetric and filament analysis module based on rigorous morphometric criteria as described previously (Imaris [2, 34]). Reconstructed dendritic segments from the CA1 stratum radiatum from each group are shown in the representative images and distinct dendritic spines were classified as filopodia (magenta), long-thin (blue), mushroom (green), and stubby (red) spines (Fig. 8a, b). As illustrated by the data, ^4^He irradiation or PLX5622 treatment did not affect the yields of either filopodia, long-thin or stubby spines 4 weeks following exposure. Interestingly, ^4^He irradiation had a significant effect on the number of mushroom spines. Two-way ANOVA indicated significant interactions for radiation and PLX5622 treatment effects on mushroom spine number (*F*_(1, 12)_ = 26.1, *P* = 0.0003 and *F*_(1, 12)_ = 30.91, *P* = 0.0001, respectively). ^4^He irradiation lead to an approximately 50% reduction in the number of mushroom spines (0 Gy + Con chow vs. 30 cGy + Con chow, *P* = 0.025). Conversely, treatment of either control or ^4^He-irradiated mice using PLX5622 induced a significant increase in mushroom spines (*P* < 0.001, 0 cGy + PLX5622 vs. 30 cGy + Con chow; *P* < 0.03, 30 Gy + PLX5622 vs. 30 cGy + Con chow). These data show that spines of defined maturation stages might exhibit differential susceptibility to space irradiation or microglia depletion.

**Fig. 9:**
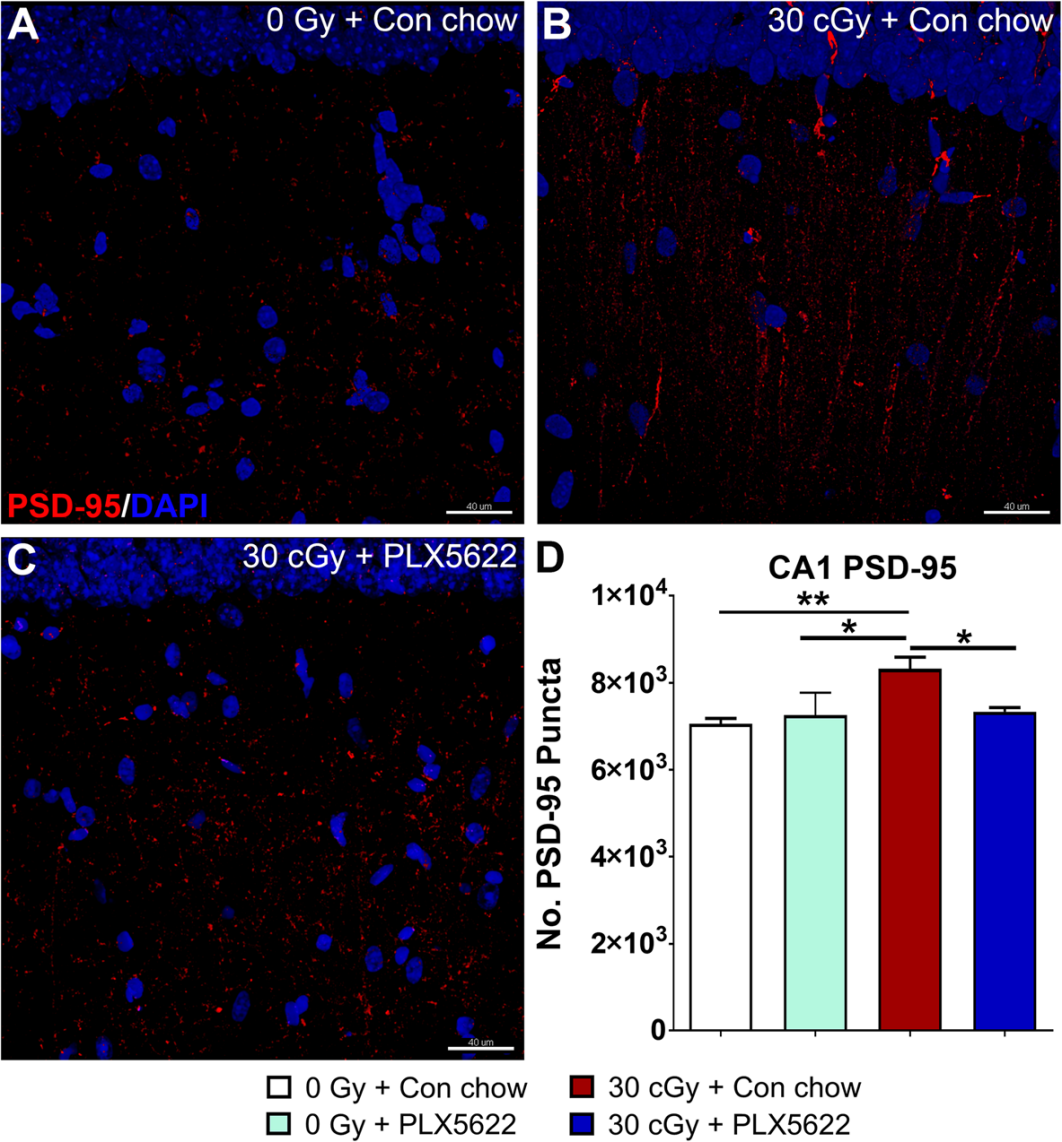
PLX5622 treatment normalizes PSD-95 expression following ^4^He irradiation. **a–c** Exposure to ^4^He irradiation (30 cGy + Con chow) leads to increased expression of PSD-95 puncta (red, nuclear counter stain, DAPI, blue) in the CA1 stratum radiatum compared to controls (*p < 0.05, **p < 0.01; 0 Gy + PLX5622 and 0 Gy + Con chow, respectively). Treatment with PLX5622 reduced PSD-95 expression in the ^4^He-irradiated brain (*p < 0.05). Data are presented as mean ± SEM (*n* = 4–6 per group). *p* values are derived from two-way ANOVA and Bonferroni’s multiple comparisons test. *p < 0.05 and **p < 0.01 compared with the 30 cGy + Con chow group. Scale bar, 40 μm, **a–c**

The effect of ^4^He and PLX5622 treatment was also evaluated in the CA1 stratum radiatum for the expression of synaptic protein PSD-95 (Fig. 9). We found a significant interaction for the radiation effect for the immunoreactivity of PSD-95^+^ puncta (*F*_(1, 16)_ = 10.51, *P* = 0.005, two-way ANOVA). Exposure to ^4^He particles lead to a significant increase in the PSD-95 puncta compared to unirradiated controls (*P* = 0.002, 0 Gy + Con chow vs. 30 cGy + Con chow) whereas treatment with PLX5622 reduced the radiation-induced elevation in PSD-95 (*P* = 0.03, 30 cGy + Con chow vs. 30 cGy + PLX5622). These data indicate that despite of marginal impact on the dendritic structure, exposure to space irradiation altered spine morphology and synaptic protein parameters which was remediated by PLX5622 treatment.

### Effects of ^4^He irradiation and PLX5622 treatment on intrinsic properties, excitability, and connectivity of neurons

To determine the potential mechanisms underlying these long-term cognitive deficits, changes in neuronal excitability properties in the perirhinal cortex (PRC) and the connectivity between the PRC and the hippocampus were measured in acute brain slices that were prepared to maximize the preservation of the connectivity between the two areas [42–45], as we had done in a previous study [3]. In the PRC, two major types of excitatory principal cells exist: the regular spiking principal cells (RSPCs) and late spiking principal cells (LSPCs, [46, 47]). The RSPCs are the majority (> 80%) of the principal cells in both superficial and deep layers of PRC. Hippocampal excitatory efferents to PRC originate in the CA1 area and target mainly deep layers (V–VI) [43, 44]. Therefore, the electrophysiology experiments were designed specifically to test the intrinsic properties of RSPCs, the incoming spontaneously occurring excitatory synaptic inputs (which likely originate from both within PRC and extra-PRC sources), and specifically the excitatory synaptic connections between CA1 and RSPCs in layer V-VI of the PRC [3].

Our data showed that the firing properties of RSPCs were not altered as a result of either ^4^He irradiation or PLX5622 treatment compared to 0 Gy + Con chow (Supplementary Figure 4A, Supplementary Table S5). In contrast, ^4^He irradiation with or without PLX5622 altered some of the intrinsic cellular excitability properties compared to controls (input resistance, *R*_in_ and resting membrane potential, V_rest_) Specifically, RSPCs showed increased *R*_in_ and hyperpolarized *V*_rest_ 1-month post-irradiation (Supplementary Figure 4B-C, Supplementary Table S5). The results without PLX5622 robustly replicated our previous findings that ^4^He irradiation increases *R*_in_ and hyperpolarizes *V*_rest_ compared to 0 cG controls [3], whereas the data with PLX5622 showed that PLX5622 had no effect on either the control (0 Gy) or the post-irradiated (i.e., altered) intrinsic excitability properties.

The properties of the excitatory synaptic inputs were assessed using spontaneously occurring miniature excitatory postsynaptic currents recorded from principal cells (mEPSCs, as in [3]). Our data showed a significant reduction in amplitude and frequency of mEPSCs in RSPCs in mice that were ^4^He irradiated with or without PLX5622 treatment compared to controls (Supplementary Figure 5A–C, Supplementary Table S6), whereas mEPSC decay time was not altered by irradiation (Supplementary Figure 5D, Supplementary Table S6). Similar to what was described above for the intrinsic properties, these results without PLX5622 replicated our previous findings that ^4^He irradiation decreases the amplitude and frequency of mEPSCs compared to 0 Gy controls [3], indicating disturbed excitatory glutamatergic fast synaptic transmission in the PRC after irradiation. Interestingly, PLX5622 caused a significant increase in mEPSC frequency in control (0 Gy) animals (compare black and red bars in Supplementary Figure 5C), likely associated with the lack of excitatory synapse (e.g. on spines) removal in the PLX5622-treated animals by the normally present active microglia. Note that the latter effect of PLX5622 on mEPSC frequency in controls also served as an effective positive control indicating that the drug was indeed active in these mice used for the electrophysiology experiments (Supplementary Figures 4 and 5). However, similarly to what was observed with the lack of a mitigating effect of PLX5622 treatment on irradiation-induced changes in intrinsic properties (Supplementary Figure 4), PLX5622 did not rescue the irradiation-induced decreases in mEPSC amplitude and frequency in the PRC (Supplementary Figure 5A–D).

In addition, in order to measure the synaptic functional connectivity between the CA1 and PRC, we placed a stimulating electrode in the CA1 hippocampal principal cell layer and recorded the intracellular responses in RSPCs in the PRC (as in [3]), in the form of evoked excitatory postsynaptic currents (eEPSCs). The probability of eliciting a postsynaptic response in PRC cells after hippocampal stimulation was measured as the percent connection probability and the data showed that 1 month after ^4^He irradiation, the functional connectivity between CA1 and RSPCs was significantly reduced irrespective of PLX5622 administration (Supplementary Figure 5E, F; Supplementary Table S7). Altogether, our electrophysiological results confirmed our previous results [3] that ^4^He particle exposure caused plasticity in the cellular and synaptic properties of the PRC circuit, while also leading to a breakdown in the communication with the hippocampus. However, interestingly, PLX5622 did not rescue these irradiation-induced persistent changes in intrinsic and synaptic excitability, in spite of the effects of microglia depletion on cognitive changes that were observed in our behavioral experiments.

## Discussion

As NASA, and other space agencies contemplate sending humans to Mars, health concerns related to prolonged deep space radiation exposure remain a considerable uncertainty. Past findings from a number of researchers have documented ample evidence in rodent models pointing to a range of behavioral impairments following acute cosmic radiation exposures, including the ^4^He particles used in this study [4–8, 17, 48, 49]. Our past studies have shown comparable and lasting impacts (6 to 52 weeks post-irradiation) of exposure to charged particles (5 cGy and 30 cGy) on CNS function ranging from low to high LET radiation-based exposures (including ^4^He, ^16^O, and ^48^Ti ions [1–3, 50]). The functional equivalence of these past data at doses ≤ 50 cGy suggest the absence of a dose response for CNS effects. Thus, for the current study, we focused on a single dose to provide data relevant to a countermeasure for cosmic radiation-induced cognitive dysfunction. Given the extensive evidence now indicating that such exposures may prove problematic at a number of levels relevant to mission critical performance and/or longer-term neurocognitive health, studies are increasingly focused on various strategies to prevent and/or mitigate the adverse effects of whole-body cosmic radiation exposure on the CNS as well as the rest of the body.

Given this backdrop, current studies have leveraged considerable past data pointing to the adverse effects of radiation-induced neuroinflammation, and more recent data sets indicating the benefits of attenuating inflammation through the elimination of microglia. We and others have shown that prolonged administration of a CSF1R inhibitor can eliminate nearly all microglia from the adult brain [25, 29, 31]. Under these conditions, normal adult animals devoid of microglia exhibit no detectable signs of any adverse consequences on locomotion or neurocognition. Under a clinical irradiation scenario, known to elicit a range of cognitive deficits and associated neuropathology [34, 51], we have demonstrated for the first time that microglial depletion through a similar administration regimen of PLX5622 ameliorated deficits in hippocampal and cortical-dependent cognitive function [29] that was also corroborated using a fractionated irradiation model [52]. Whether microglia are dispensable for longer-term neurocognitive health or over the duration of deep space exploration is uncertain. The present paper points to defined benefits of reducing microglia levels following cosmic radiation exposure.

Current data demonstrates that CSF1R blockade can ameliorate ^4^He irradiation-induced behavioral decrements on four out of five cognitive function tasks involving hippocampal and cortical based learning and memory. On the other hand, ^4^He particle exposure did not affect locomotion, or induce anxiety or depression behaviors (EPM and FST). A past study, using two behavior tasks (NOR and EPM) showed equal effectiveness of short-term PLX5622 treatment to ameliorate radiation-induced impairments on the NOR task [18]. Our past work and the work of others also demonstrated no impact of CSF1R blockade on anxiety 3 months after exposure to ^4^He particles [2, 18]. While reduced microglial levels were beneficial to recognition, episodic and working memory, perhaps the most critical finding in the context of deep space exploration, where complex activities must be executed under the utmost autonomy, was the ability of CSF1R blockade to resolve deficits on FE to maintain cognitive flexibility. All the groups (control or irradiated with or without PLX5622) showed elevated freezing after exposure to tone-shock pairing indicating intact conditioning response. However, during the subsequent fear extinction training, ^4^He-irradiated mice failed to extinguish from the conditioning response indicating compromised memory consolidation. In contrast, PLX5622 treatment of the irradiated animals was able to restore memory consolidation. Over the duration of a mission to Mars, astronauts will likely encounter a variety of unanticipated scenarios requiring adaptive learning, abstract reasoning, and the ability to make rapid decisions. Maintaining cognitive flexibility will be critical under these circumstances to ensure optimal performance and mission outcomes.

The robust reversal of adverse neurocognitive outcomes associated with low dose ^4^He particle exposure afforded by CSF1R blockade suggested that radiation-induced inflammatory pathologies were also affected. Prior work, using PFC tissue flow cytometry to analyze microglial depletion in the ^4^He exposed brain, found that repopulated microglia had reduced phagocytic activity that could impact radiation-induced inflammatory changes and synaptic protein expression [18]. Our past studies have shown the persistent impact of an oxidative environment on neural stem cells (in vitro), hippocampal neuron structure and cognitive function [10, 14, 53–55]. Radiation-induced elevation in oxidative and nitrosative stress could also trigger microglial activation, although, we have not measured redox parameters in the current study. Nonetheless, the possible direct impact of charged particle exposure on microglia cannot be denied. For this study we used middle-aged mice (6+ months) to match the approximate age of astronauts. After completion of behavior, these mice were, on average 7–8 months of age. A study using C57Bl6/J mice has shown about 60–80% reduction in the hippocampal neurogenesis at 10 months of age compared to 2-month-old young mice [56]. Such a dramatic decline in neurogenesis in the C57 black mice with aging may confound the analysis of either a radiation effect or a drug treatment effect. Thus, our study was concentrated on the mature neuronal circuits and cognitive function. Moreover, we did not see any impact of either charged particles or PLX5622 treatment on the oligodendrocyte progenitor cell number. On the other hand, PLX5622 treatment led to a marginal decrease in the astrocyte immunoreactivity in the ^4^He-irradiated brain. Taken together, PLX5622 treatment and cosmic irradiation has a major impact on microglial activation. A past study using a similar cosmic irradiation model has shown that exposure to ^4^He irradiation did not change either number of microglia or peripherally derived macrophage/monocyte numbers [18]. Transient treatment with a CSF1R inhibitor (2 weeks) depleted both macrophages and microglia. Importantly, both, ours and past study, show increased expression of reactive microglia (CD68, LAMP-1, and CD206) in the Helium-irradiated brains. Moreover, given the low cosmic irradiation dose (30 cGy) and the post-IRR times of analysis (6–8 weeks), persistent macrophage infiltration is highly unlikely. To scrutinize neuron morphologic endpoints, we undertook extensive analyses of hippocampal CA1 neuronal morphologic parameters, representing a region of the brain different from much of our past work analyzing the effects of space radiation on prefrontal cortex neurons [2]. These new data indicate that CA1 neurons exhibit reduced sensitivity to ^4^He particle irradiation, as overt changes in dendritic complexity were not found. This is in contrast to changes found in the mPFC over similar (6 weeks) and longer (15 weeks) post-irradiation intervals, where the same low dose exposures using heavier ions (^16^O, ^48^Ti) caused more robust deterioration of neuronal structure [1, 2]. Further analyses of dendritic spine parameters did not reveal radiation effects. Neuronal excitability and function is dependent upon the formation and maturation of dendritic spines that integrates the synaptic landscape. Reductions in the number and volume of dendritic spines has been reported in several neuropsychological and neurodegenerative disorders [36, 57–60]. Our data show that exposure to ^4^He drastically reduced the number of mushroom spines with marginal impact on immature (filopodia, lont/thin) and stubby spines. Mushroom spines are considered to be more mature and functionally stronger spines with expression of AMPA receptors [61, 62]. Our past report showed a significant reduction in the number of filopodia, long, and mushroom spine types 12–14 weeks after exposure to ^16^O and ^48^Ti ions [2]. Mushroom spines also have larger post-synaptic density and thereby provide higher synaptic efficacy. Radiation-induced reductions in the number of mushroom spines may serve as one of the major contributory factors to the loss of neuronal function and cognitive impairment. Moreover, microglia actively regulate the synaptic and pre-synaptic environment by pruning the dendritic spines [63] and ^4^He radiation-induced elevated microglial activation, providing a plausible explanation for the loss of mature mushroom spines in the CA1 circuitry. Microglia elimination significantly increased the number of mushroom spines in both control and ^4^He-irradiated animals, thereby providing compelling evidence for the disruptive role of radiation-induced neuroinflammation. Lastly, and in agreement with our past work and other reports [1, 2], ^4^He particle exposures increased PSD-95 levels, possibly reflecting an increased expression and/or redistribution of this critical synaptic assembly factor from the spine head to the dendritic shaft. The ability of CSF1R blockade to re-normalize levels of PSD-95 puncta suggests a role for microglia in modulating the structural plasticity of dendritic synapses following irradiation [64, 65].

CSF1R inhibitors are currently under clinical trials to determine their safety and efficacy in the context of cancer treatments [66]. Studies using viral infection-mediated inflammation in adult animals showed detrimental physiological effects of PLX5622 treatment prior to virus infection on the CNS immune response [67], axonal damage, and survival [68] showing a cautionary indication. Similarly, depletion of microglia prior to neuronal insult aggravated the injury, whereas microglia elimination following the neuronal injury promoted recovery [27]. These studies indicate that microglia elimination is not beneficial prior to a major CNS insult. However, a series of mechanistic studies by Green and colleagues have shown neuroprotective effects of CSF1R inhibition in various neurodegeneration models [27, 30, 69]. For example, sustained microglia elimination for the period of seven months in the 5xFAD mouse prevented plaque formation whereas re-population of microglia upon CSF1R inhibitor withdrawal led to robust plaque formation [69]. No adverse effects of PLX5622 treatment were observed on neural stem and oligo-progenitor cell proliferation and differentiation [70, 71]. Moreover, treatment with PLX5622 showed neurocognitive benefits and attenuation of neuroinflammation in a chemobrain model [72]. Microglia depletion rescued most radiation-induced cognitive changes observed in our behavioral tests, reduced microglial activation, and restored mature dendritic spines. PLX5622 treatment did not, however, reverse radiation-induced alterations in intrinsic excitability and excitatory synaptic transmission properties in the PRC neurons. These latter results suggest that microglial depletion may not resolve all radiation-induced persistent changes in the CNS. Future studies will be needed to determine the reason that PLX5622 did not mitigate the radiation-induced changes in intrinsic and synaptic excitability but could effectively ameliorate the effects of irradiation on PSD-95 protein levels and mushroom spine numbers. Several factors may affect the intrinsic excitability or excitatory synaptic transmission properties of neurons. In addition, morphological and electrophysiological measurements likely have different detection sensitivity and may not necessarily sample completely overlapping biological events and processes. For example, mEPSCs may have, at least in part, originated from synapses located on spines that may not perfectly correspond to the anatomical definition of mushroom spines. Importantly, our data did corroborate our past findings showing the long-term impacts of ^4^He irradiation on salient properties of CA1 and PRC neurons (RSPCs). Our past data also showed the disruptive effects of radiation-induced oxidative stress and inflammation on neurotransmission [10, 14], and selective changes in synaptic function that regulate GABA release in the hippocampal CA1 microcircuit [20]. Field recording studies by Vlkolinsky and colleagues have shown the impact of various LET and fluencies of charged particles including ^28^Si, ^56^Fe, and protons on EPSC and population spikes for CA1 neurons [73–75]. Future studies will need to investigate the mechanistic underpinnings of the apparently differential effects of microglial depletion on radiation-induced changes as observed in behavioral tests and alterations in basic electrophysiological properties in the PRC.

## Conclusions

Taken together, the results of this study demonstrate the positive neurocognitive benefits of microglia elimination in the brain subjected to cosmic radiation exposure. The fact that CSF1R expression is specific to microglia and induces no apparent side effects makes this strategy an attractive intervention for ameliorating the adverse effects of radiation exposure on cognition. How permanent (this study) or transient [18] CSF1R inhibition affects cognitive function at longer-term post-irradiation time, and how temporally optimize windows of time for microglial depletion after irradiation remain to be determined. While further work is required to define administration schedules suitable to intervene against the adverse effects of space radiation exposure, current and past data [18] suggest that approaches implementing CSF1R blockade to eliminate microglia are poised to provide considerable benefit to the irradiated CNS.

## Supplementary information

**Additional file 1: Figure S1.**^4^He irradiation or PLX5622 treatment did not alter neuron dendritic parameters. **a-d** Quantification of the number of dendritic branches and branch points from the dentate gyrus (DG) granule cell layer (GCL) neuron and CA1 pyramidal neuron did not show a significant difference at 4-week post-irradiation between the control (0 Gy) and ^4^He irradiated (30 cGy) mice treated with PLX5622. Data are presented as Mean ± SEM (n = 4/group).

**Additional file 2: Figure S2.** Effect of ^4^He particle exposure or PLX5622 treatment on the spine density parameters. **a**-**b** Quantification of spine density in the GCL neuron showed a significant decrease in the following ^4^He irradiation (**p<0.01 vs. 30 cGy + Con chow; ^+^*p*<0.05 vs. 0 Gy + PLX5622). Irradiated mice receiving PLX5622 treatment show a significant improvement in the spine density at 4-week post-treatment (*p<0.05 vs. 30 cGy + Con chow). Data are presented as Mean ± SEM (n = 4/group). *P* values are derived from ANOVA and Bonferroni’s multiple comparisons test. *p<0.05 and **p<0.01 compared with the 30 cGy + Con chow group; ^+^p<0.05 compared with 0 Gy + PLX5622 group.

**Additional file 3: Figure S3.** PLX5622 treatment or ^4^He irradiation did not alter spine volume. **a** Representative deconvoluted reconstruction of eGFP^+^ CA1 pyramidal neuron dendrites (green) and spines (blue). **b** Quantification of the spine volume from the dentate gyrus (DG) granule cell layer (GCL) neuron and CA1 pyramidal neuron show a trend of radiation-induced reduction and PLX5622-mediated recovery, however, statistically indistinguishable. Data are presented as Mean ± SEM (n = 4/group). Scale bar, 5 μm, **a**.

**Additional file 4: Figure S4.** Alteration of the intrinsic properties of perirhinal cortex regular spiking principal cells (RSPCs) after ^4^He particle irradiation, and the lack of rescue by PLX5622. **a** Representative whole cell current-clamp recordings of RSPCs from control (0 Gy +Con, black), control + PLX5622 (red), irradiated (30 cGy + Con chow, purple) and 30 cGy + PLX5622 (blue) mice. **b** Bar graphs of input resistances (Rin) of RSPCs showing the effect of ^4^He exposure 1 month post-irradiation. **c** Bar graphs of resting membrane potentials (Vrest) of RSPCs showing the hyperpolarizing effects of ^4^He exposure 1 month after irradiation (Suppl. Table S5). **p<0.01 by one-way ANOVA followed by Tuckey’s post-hoc test. Data are expressed as the mean ± SEM (as in Suppl. Table S5).

**Additional file 5: Figure S5.** Perirhinal cortex excitability and connectivity is impaired in regular spiking principal cell (RSPCs) in mice irradiated with ^4^He, and these effects are unaltered by PLX5622. **a** Representative miniature EPSCs (mESPCs) recordings in RSPCs from control (0 Gy +Con, black), control + PLX5622 (red), irradiated (30 cGy + Con chow, purple) and 30 cGy + PLX5622 (blue) mice. **b** amplitude, **c** frequency and **d** decay time showing the effect of ^4^He exposure 1 month time after irradiation (Suppl. Table S6). Note the increase in mEPSC frequency in control animals after PLX5622, indicating that the drug was active under these conditions (positive control). **e** Representative electrical stimulation-evoked EPSCs (eEPSCs) recordings in RSPCs and f bar graphs of the connection probability between CA1 and RSPCs showing that ^4^He exposure significantly reduced the CA1-evoked EPSC amplitude and connection probability 1 month after irradiation (x/y connected for each group; Suppl. Table S7), *p<0.05, **p<0.001 by one-way ANOVA followed by Tuckey’s post-hoc test (Panels **b**-**d**) and **p< 0.01 by chi-square test (Panel **f**). Data are expressed as mean ± SEM or as single value (panels **b**-**d** and as (number of positive connections/total trials)*100 ) (panel **f**; as in Suppl. Tables S6 and S7).

**Additional file 6: Table S1.** NOR task: total time spent (sec) exploring both objects.

**Additional file 7: Table S2.** OiP task: total time spent (sec) exploring both objects.

**Additional file 8: Table S3.** TO task: total time spent (sec) exploring both objects.

**Additional file 9: Table S4.** NPR task: total time spent (sec) exploring both objects.

**Additional file 10: Table S5.** Regular spiking principal cells (RSPCs) intrinsic properties.

**Additional file 11: Table S6.** Properties of miniature EPSCs (mEPSCs) recorded in RSPCs.

**Additional file 12: Table S7.** Properties of CA1-evoked EPSCs in RSPCs and connection probability.

## Data Availability

All the data used in this manuscript are available on request.

## Abbreviations

^16^O: Oxygen particles
^28^Si: Silicon particles
^48^Ti: Titanium particles
^4^He: Helium particles
^56^Fe: Iron particles
ANOVA: Analysis of variance
BNL: Brookhaven National Laboratory
C57Bl/6J: In-bred black mouse strain
CA1: Cornu ammonis 1
CD68: Cluster of differentiation 68 protein
CNS: Central nervous system
CS: Conditional stimulus
CSF1R: Colony stimulating factor-1 receptor
DI: Discrimination index
eEPSCs: Evoked excitatory postsynaptic currents
EGFP: Enhanced green fluorescent protein
EPM: Elevated plus maze
FE: Fear extinction
FST: Forced swim test
GABA: Gamma amino butyric acid
GCL: Granule cell layer
IBA1: Ionized calcium-binding adapter molecule 1 protein
LSPCs: Late spiking principal cells
mEPSCs: Miniature excitatory postsynaptic currents
NASA: National Aeronautics and Space Administration
NOR: Novel object recognition
NPR: Novel place recognition
OiP: Object in place
PLX5622: CSF1R inhibitor molecule
PRC: Perirhinal cortex
PSD-95: Postsynaptic density protein 95
RSPCs: Regular spiking principal cells
TBS: Tri-buffered saline
Thy1: Thy-1 cell surface antigen
TO: Temporal order
US: Unconditional stimulus
