# Supplementary figures and images for: Mitigation of helium irradiation-induced brain injury by microglia depletion
Source: J Neuroinflammation. 2020 May 19;17:159. doi: 10.1186/s12974-020-01790-9 (PMC7236926; doi:10.1186/s12974-020-01790-9)

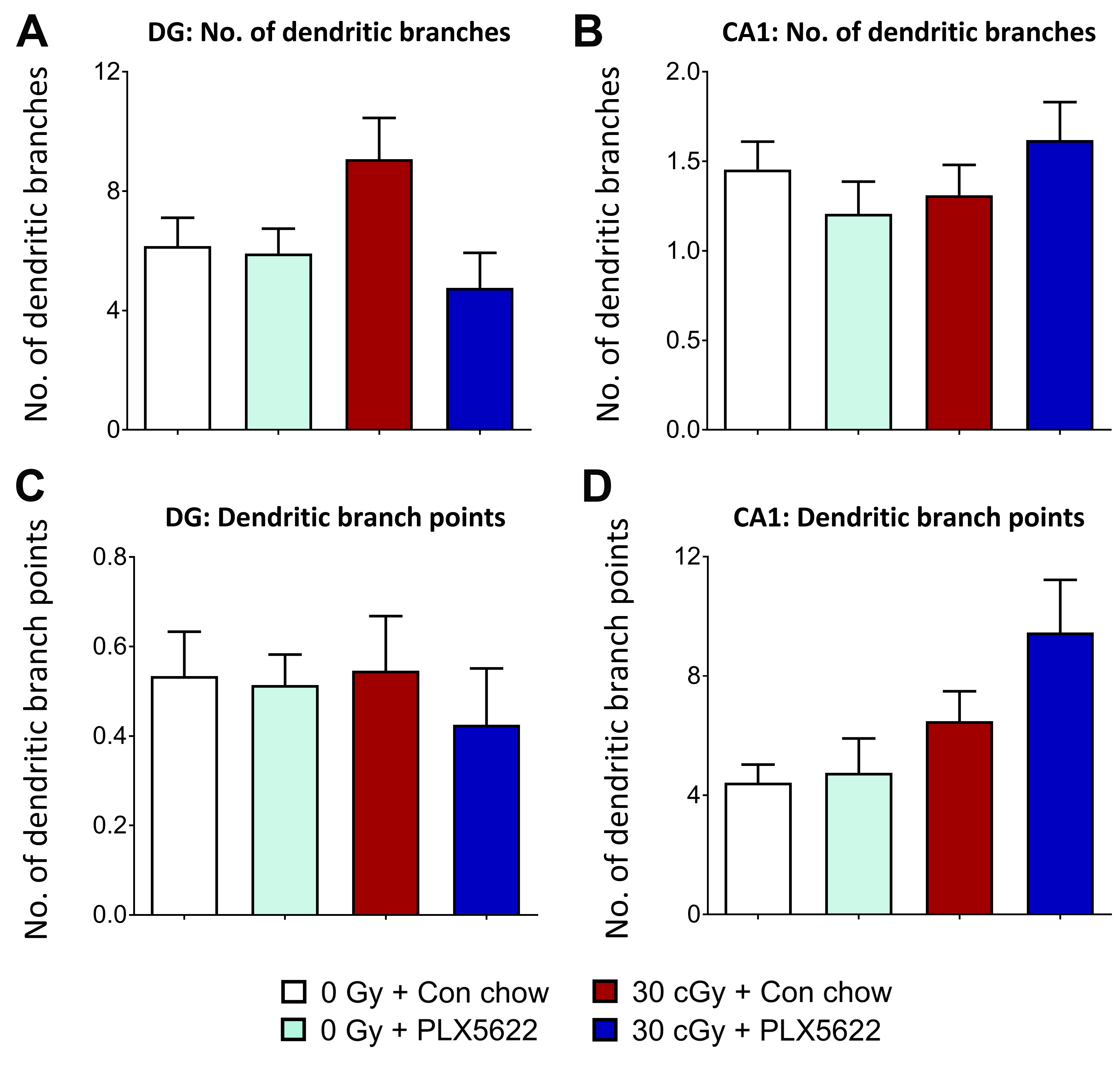

Supplement: Supplementary file 1 — Additional file 1: Figure S1.4He irradiation or PLX5622 treatment did not alter neuron dendritic parameters. a-d Quantification of the number of dendritic branches and branch points from the dentate gyrus (DG) granule cell layer (GCL) neuron and CA1 pyramidal neuron did not show a significant difference at 4-week post-irradiation between the control (0 Gy) and 4He irradiated (30 cGy) mice treated with PLX5622. Data are presented as Mean ± SEM (n = 4/group). [file 12974_2020_1790_MOESM1_ESM.tif]

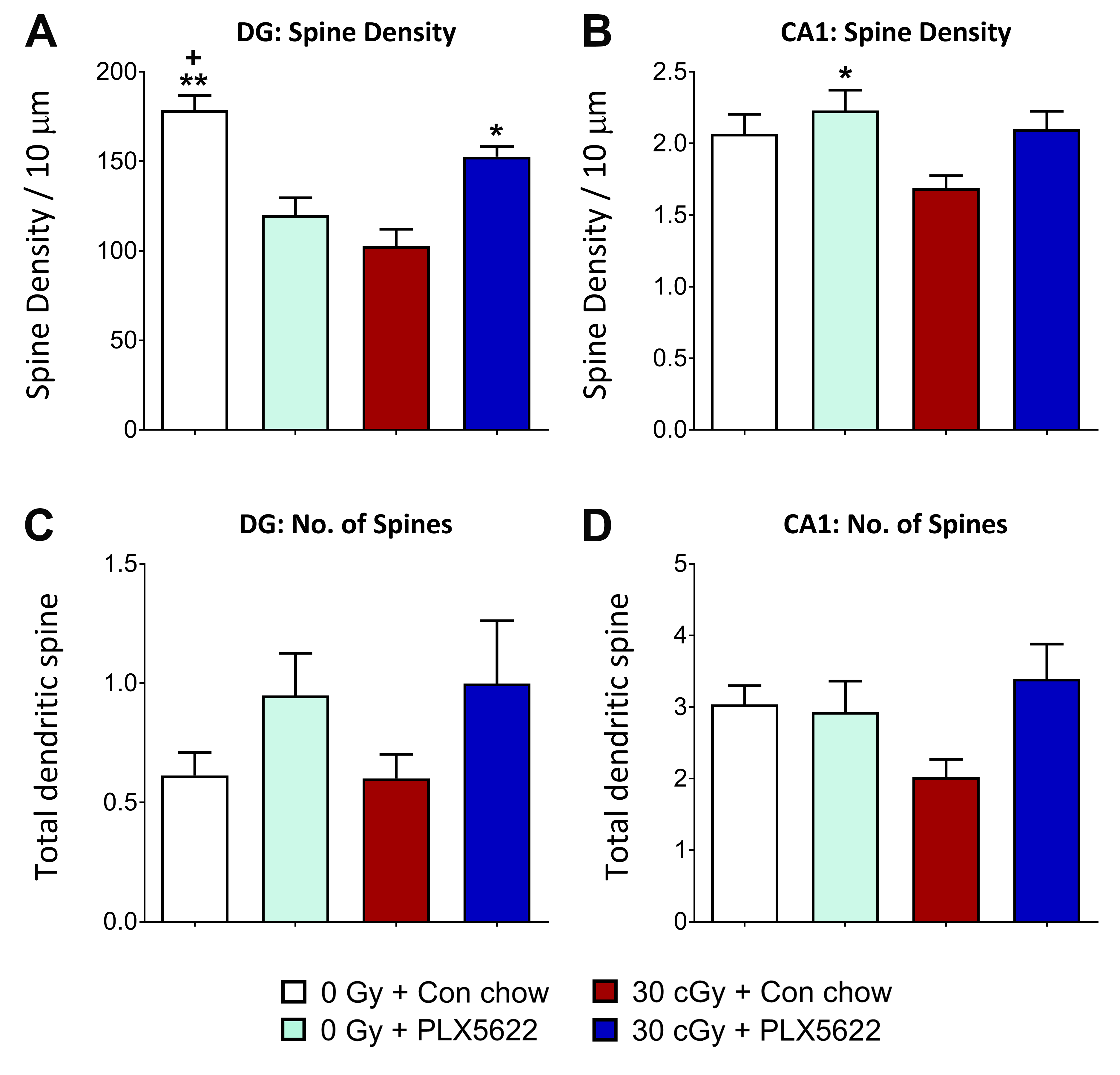

Supplement: Supplementary file 2 — Additional file 2: Figure S2. Effect of 4He particle exposure or PLX5622 treatment on the spine density parameters. a-b Quantification of spine density in the GCL neuron showed a significant decrease in the following 4He irradiation (**p<0.01 vs. 30 cGy + Con chow; +p<0.05 vs. 0 Gy + PLX5622). Irradiated mice receiving PLX5622 treatment show a significant improvement in the spine density at 4-week post-treatment (*p<0.05 vs. 30 cGy + Con chow). Data are presented as Mean ± SEM (n = 4/group). P values are derived from ANOVA and Bonferroni’s multiple comparisons test. *p<0.05 and **p<0.01 compared with the 30 cGy + Con chow group; +p<0.05 compared with 0 Gy + PLX5622 group. [file 12974_2020_1790_MOESM2_ESM.tif]

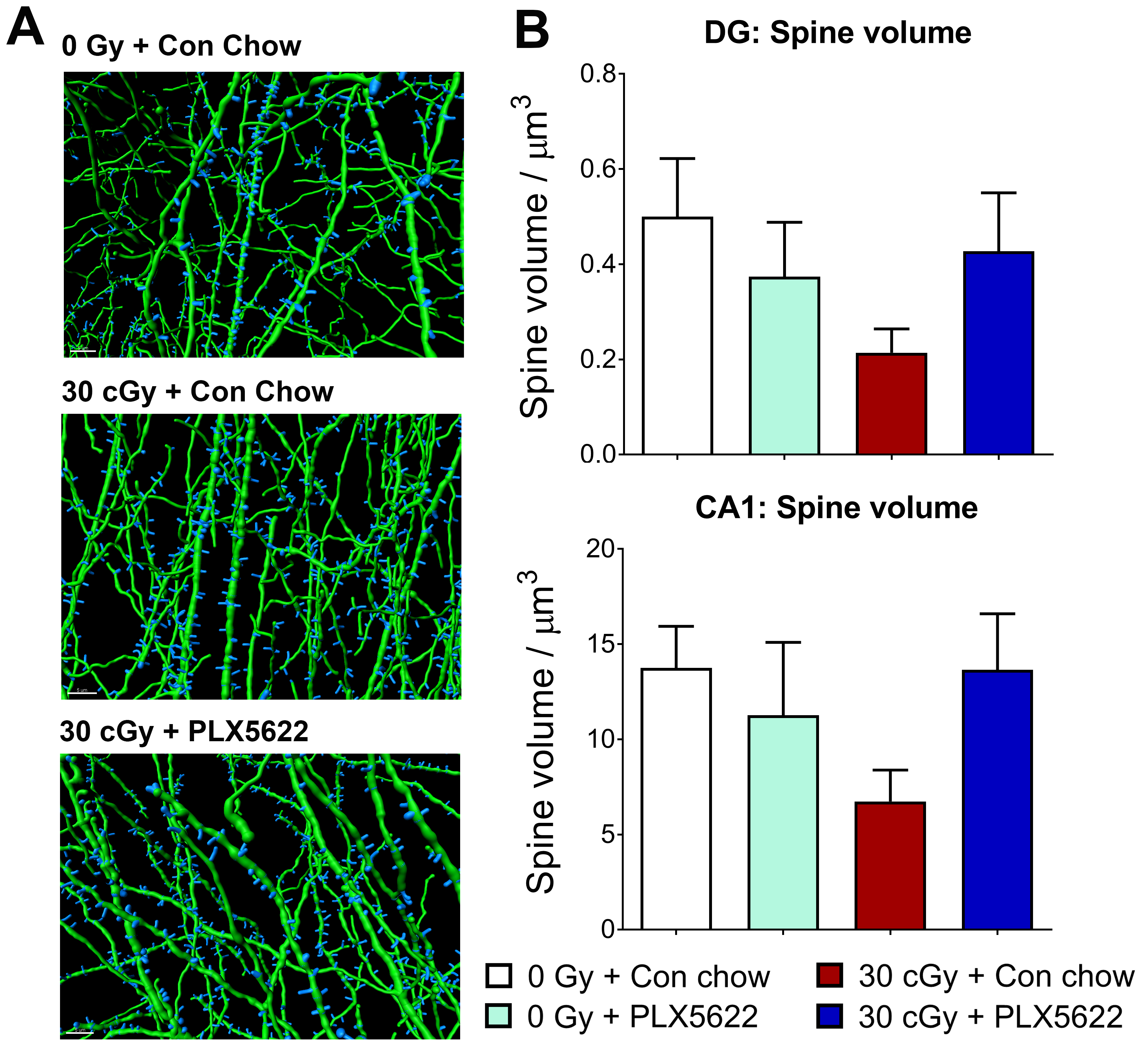

Supplement: Supplementary file 3 — Additional file 3: Figure S3. PLX5622 treatment or 4He irradiation did not alter spine volume. a Representative deconvoluted reconstruction of eGFP+ CA1 pyramidal neuron dendrites (green) and spines (blue). b Quantification of the spine volume from the dentate gyrus (DG) granule cell layer (GCL) neuron and CA1 pyramidal neuron show a trend of radiation-induced reduction and PLX5622-mediated recovery, however, statistically indistinguishable. Data are presented as Mean ± SEM (n = 4/group). Scale bar, 5 μm, a. [file 12974_2020_1790_MOESM3_ESM.tif]

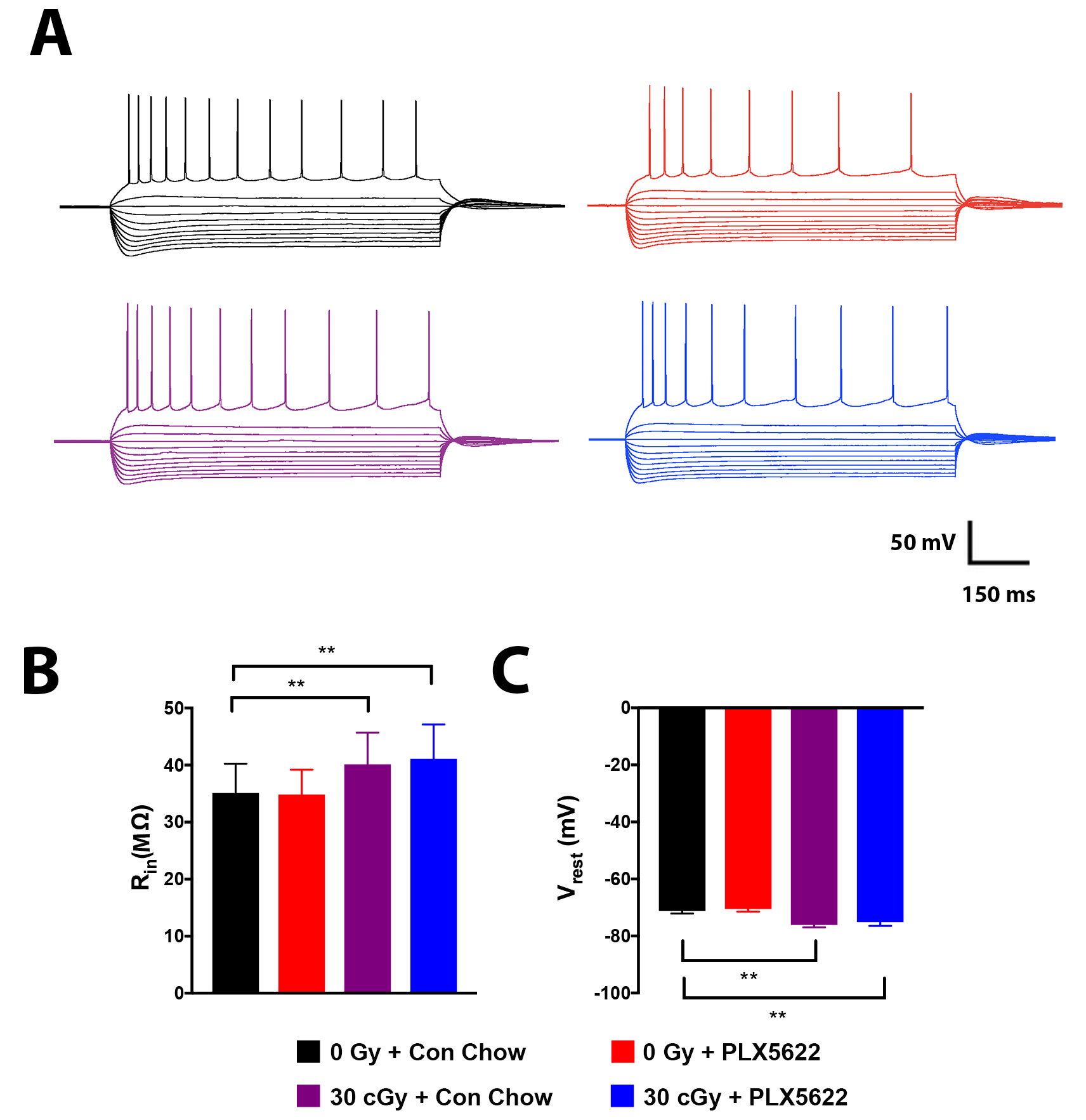

Supplement: Supplementary file 4 — Additional file 4: Figure S4. Alteration of the intrinsic properties of perirhinal cortex regular spiking principal cells (RSPCs) after 4He particle irradiation, and the lack of rescue by PLX5622. a Representative whole cell current-clamp recordings of RSPCs from control (0 Gy +Con, black), control + PLX5622 (red), irradiated (30 cGy + Con chow, purple) and 30 cGy + PLX5622 (blue) mice. b Bar graphs of input resistances (Rin) of RSPCs showing the effect of 4He exposure 1 month post-irradiation. c Bar graphs of resting membrane potentials (Vrest) of RSPCs showing the hyperpolarizing effects of 4He exposure 1 month after irradiation (Suppl. Table S5). **p<0.01 by one-way ANOVA followed by Tuckey’s post-hoc test. Data are expressed as the mean ± SEM (as in Suppl. Table S5). [file 12974_2020_1790_MOESM4_ESM.tif]

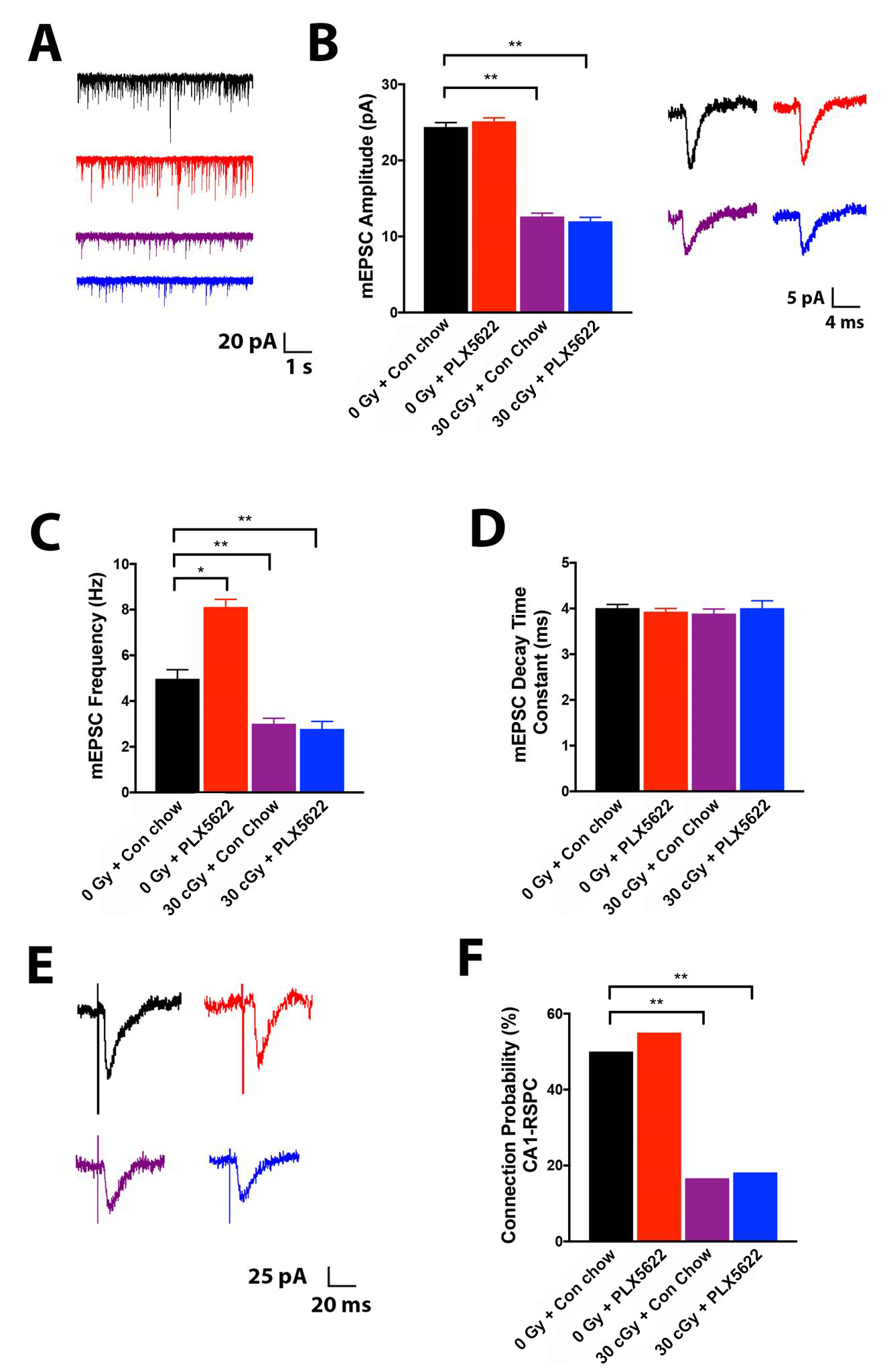

Supplement: Supplementary file 5 — Additional file 5: Figure S5. Perirhinal cortex excitability and connectivity is impaired in regular spiking principal cell (RSPCs) in mice irradiated with 4He, and these effects are unaltered by PLX5622. a Representative miniature EPSCs (mESPCs) recordings in RSPCs from control (0 Gy +Con, black), control + PLX5622 (red), irradiated (30 cGy + Con chow, purple) and 30 cGy + PLX5622 (blue) mice. b amplitude, c frequency and d decay time showing the effect of 4He exposure 1 month time after irradiation (Suppl. Table S6). Note the increase in mEPSC frequency in control animals after PLX5622, indicating that the drug was active under these conditions (positive control). e Representative electrical stimulation-evoked EPSCs (eEPSCs) recordings in RSPCs and f bar graphs of the connection probability between CA1 and RSPCs showing that 4He exposure significantly reduced the CA1-evoked EPSC amplitude and connection probability 1 month after irradiation (x/y connected for each group; Suppl. Table S7), *p<0.05, **p<0.001 by one-way ANOVA followed by Tuckey’s post-hoc test (Panels b-d) and **p< 0.01 by chi-square test (Panel f). Data are expressed as mean ± SEM or as single value (panels b-d and as (number of positive connections/total trials)*100 ) (panel f; as in Suppl. Tables S6 and S7). [file 12974_2020_1790_MOESM5_ESM.tif]
