# Supplementary material for: Mitigation of helium irradiation-induced brain injury by microglia depletion
Source: J Neuroinflammation. 2020 May 19;17:159. doi: 10.1186/s12974-020-01790-9 (PMC7236926; doi:10.1186/s12974-020-01790-9)
Supplement: Supplementary file 7 — Additional file 7: Table S2. OiP task: total time spent (sec) exploring both objects. [file 12974_2020_1790_MOESM7_ESM.docx]

**Suppl. Table 2.** OiP task: total time spent (sec) exploring both objects

| **Total time spent exploring both objects** | **Mean** | **SEM** | **N** |
| --- | --- | --- | --- |
| 0 Gy + Con chow | 38.588 | 3.542 | 8 |
| 0 Gy + PLX5622 | 25.190 | 3.890 | 8 |
| 30 cGy + Con chow | 33.659 | 2.753 | 8 |
| 30 cGy + PLX5622 | 23.370 | 4.471 | 8 |
