# Supplementary material for: Mitigation of helium irradiation-induced brain injury by microglia depletion
Source: J Neuroinflammation. 2020 May 19;17:159. doi: 10.1186/s12974-020-01790-9 (PMC7236926; doi:10.1186/s12974-020-01790-9)
Supplement: Supplementary file 8 — Additional file 8: Table S3. TO task: total time spent (sec) exploring both objects. [file 12974_2020_1790_MOESM8_ESM.docx]

**Suppl. Table 3.** TO task: total time spent (sec) exploring both objects

| **Total time spent exploring both objects** | **Mean** | **SEM** | **N** |
| --- | --- | --- | --- |
| 0 Gy + Con chow | 15.036 | 3.164 | 8 |
| 0 Gy + PLX5622 | 10.821 | 1.803 | 8 |
| 30 cGy + Con chow | 17.250 | 1.847 | 8 |
| 30 cGy + PLX5622 | 9.765 | 1.145 | 8 |
