# Supplementary material for: Mitigation of helium irradiation-induced brain injury by microglia depletion
Source: J Neuroinflammation. 2020 May 19;17:159. doi: 10.1186/s12974-020-01790-9 (PMC7236926; doi:10.1186/s12974-020-01790-9)
Supplement: Supplementary file 9 — Additional file 9: Table S4. NPR task: total time spent (sec) exploring both objects. [file 12974_2020_1790_MOESM9_ESM.docx]

**Suppl. Table 4.** NPR task: total time spent (sec) exploring both objects

| **Total time spent exploring both objects** | **Mean** | **SEM** | **N** |
| --- | --- | --- | --- |
| 0 Gy + Con chow | 38.738 | 3.748 | 10 |
| 0 Gy + PLX5622 | 43.111 | 4.694 | 10 |
| 30 cGy + Con chow | 41.320 | 4.541 | 10 |
| 30 cGy + PLX5622 | 31.686 | 3.130 | 10 |
