# Supplementary material for: Mitigation of helium irradiation-induced brain injury by microglia depletion
Source: J Neuroinflammation. 2020 May 19;17:159. doi: 10.1186/s12974-020-01790-9 (PMC7236926; doi:10.1186/s12974-020-01790-9)
Supplement: Supplementary file 10 — Additional file 10: Table S5. Regular spiking principal cells (RSPCs) intrinsic properties. [file 12974_2020_1790_MOESM10_ESM.docx]

**Supplemental Table 5:** Regular spiking principal cells (RSPCs) intrinsic properties

| **^4^He (cGy)** | **Diet** | **V_rest_**  **(mV)** | **R_in_**  **(MΩ)** | **τ, ms** | **Maximum Firing Rate, s^−1^** | **Spike Duration, ms** | **Spike Rise, ms** | **Spike Half-Width, ms** | **AP amplitude, mV** | **AP Threshold** | **Sag Ratio** |
| --- | --- | --- | --- | --- | --- | --- | --- | --- | --- | --- | --- |
|  |  |  |  |  |  |  |  |  |  |  |  |
| 0 | Con chow | -71.22 ± 0.91 | 35.12 ± 5.12 | 35 ± 4 | 33 ± 5 | 3.04 ± 0.11 | 1.39 ± 0.04 | 0.77 ± 0.08 | 99 ± 4 | 31.4 ± 3.8 | 0.84 ± 0.05 |
|  | PLX5622 | -70.51 ± 0.94 | 34.84 ± 4.37 | 30 ± 5 | 32 ± 6 | 3.57 ± 0.06 | 1.39 ± 0.08 | 0.72 ± 0.09 | 97 ± 4 | 31.6 ± 4.1 | 0.80 ± 0.07 |
| 30 | Con chow | -76.12 ± 0.84** | 40.14 ± 5.56** | 29 ± 7 | 36 ± 7 | 2.84 ± 0.07 | 1.41 ± 0.05 | 0.71 ± 0.05 | 96 ± 5 | 29.4 ± 3.9 | 0.79 ± 0.10 |
|  | PLX5622 | -75.12 ± 1.32** | 41.12 ± 6.01** | 30 ± 7 | 32 ± 4 | 2.95 ± 0.06 | 1.39 ± 0.04 | 0.75 ± 0.10 | 93 ± 6 | 31.1 ± 4.1 | 0.81 ± 0.07 |

Data are reported as mean ± SEM; 0 Gy + Con chow n=10, 0 Gy + PLX5622 n=11; 30 cGy + Con chow n=10, 30 cGy + PLX5622 n=12; ** p<0.001 vs. 0 cGy + Con chow by two-way ANOVA followed by Tukey’s post hoc test.
