# Supplementary material for: Mitigation of helium irradiation-induced brain injury by microglia depletion
Source: J Neuroinflammation. 2020 May 19;17:159. doi: 10.1186/s12974-020-01790-9 (PMC7236926; doi:10.1186/s12974-020-01790-9)
Supplement: Supplementary file 11 — Additional file 11: Table S6. Properties of miniature EPSCs (mEPSCs) recorded in RSPCs. [file 12974_2020_1790_MOESM11_ESM.docx]

**Supplemental Table 6:** Properties of miniature EPSCs (mEPSCs) recorded in RSPCs

| **Helium Irradiation**  **(cGy)** | **Diet** | **Amplitude**  **(pA)** | **Frequency**  **(Hz)** | **Decay time**  **(ms)** |
| --- | --- | --- | --- | --- |
| 0 | Con chow | 24.37 ± 0.58 | 5.12 ± 0.39 | 4.01 ± 0.08 |
|  | PLX5622 | 25.12 ± 0.45 | 8.01 ± 0.33* | 3.93 ± 0.07 |
| 30 | Con chow | 12.61 ± 0.44** | 2.92 ± 0.24** | 3.89 ± 0.10 |
|  | PLX5622 | 11.98 ± 0.52** | 2.61 ± 0.33** | 4.01 ± 0.16 |

Data are reported as mean ± SEM; 0 Gy + Con chow n=10, 0 Gy + PLX5622 n=11; 30 cGy + Con chow Con n=10, 30 cGy + PLX5622 n=12; * p<0.05, ** p<0.001 vs. 0 cGy + Con chow by two-way ANOVA followed by Tukey’s post hoc test.
