# Supplementary material for: Mitigation of helium irradiation-induced brain injury by microglia depletion
Source: J Neuroinflammation. 2020 May 19;17:159. doi: 10.1186/s12974-020-01790-9 (PMC7236926; doi:10.1186/s12974-020-01790-9)
Supplement: Supplementary file 12 — Additional file 12: Table S7. Properties of CA1-evoked EPSCs in RSPCs and connection probability. [file 12974_2020_1790_MOESM12_ESM.docx]

**Supplemental Table 7:** Properties of CA1-evoked EPSCs in RSPCs and connection probability

| **Helium Irradiation**  **(cGy)** | **Diet** | **Amplitude**  **(pA)** | **Decay time**  **(ms)** | **Connection Probability** |
| --- | --- | --- | --- | --- |
| 0 | Con chow | 30.44 ± 0.13 | 3.97 ± 0.07 | 7/14 |
|  | PLX5622 | 32.12 ± 0.67 | 4.03 ± 0.09 | 6/11 |
| 30 | Con chow | 16.22 ± 0.53 | 4.10 ± 0.05 | 2/12 |
|  | PLX5622 | 17.87 ± 0.38 | 3.97 ± 0.12 | 2/11 |

Data are reported as mean ± SEM; 0 cGy + Con chow n=7, 0 Gy + PLX5622 n=6; 30 cGy + Con chow n=2, 30 cGy + PLX5622 n=2; Please note that due to low connection probability in the irradiated animals, the number of samples was too low to perform statistical analysis; Connection Probability: number of successes to evoke EPSCs after CA1 stimulation / number of trials)
